# Immunological memory to SARS-CoV-2 assessed for up to eight months after infection

**DOI:** 10.1101/2020.11.15.383323

**Published:** 2020-12-18

**Authors:** Jennifer M. Dan, Jose Mateus, Yu Kato, Kathryn M. Hastie, Esther Dawen Yu, Caterina E. Faliti, Alba Grifoni, Sydney I. Ramirez, Sonya Haupt, April Frazier, Catherine Nakao, Vamseedhar Rayaprolu, Stephen A. Rawlings, Bjoern Peters, Florian Krammer, Viviana Simon, Erica Ollmann Saphire, Davey M. Smith, Daniela Weiskopf, Alessandro Sette, Shane Crotty

**Affiliations:** 1 Center for Infectious Disease and Vaccine Research, La Jolla Institute for Immunology (LJI), La Jolla, CA 92037, USA; 2 Department of Medicine, University of California, San Diego (UCSD), La Jolla, CA 92037, USA; 3 Department of Medicine, Division of Infectious Diseases and Global Public Health, University of California, San Diego (UCSD), La Jolla, CA 92037, USA; 4 Department of Microbiology, Icahn School of Medicine at Mount Sinai, New York, New York; 5 Division of Infectious Diseases, Department of Medicine, Icahn School of Medicine at Mount Sinai, New York, NY 10029, USA; 6 The Global Health and Emerging Pathogens Institute, Icahn School of Medicine at Mount Sinai, New York, NY 10029, USA

## Abstract

Understanding immune memory to SARS-CoV-2 is critical for improving diagnostics and vaccines, and for assessing the likely future course of the COVID-19 pandemic. We analyzed multiple compartments of circulating immune memory to SARS-CoV-2 in 254 samples from 188 COVID-19 cases, including 43 samples at ≥ 6 months post-infection. IgG to the Spike protein was relatively stable over 6+ months. Spike-specific memory B cells were more abundant at 6 months than at 1 month post symptom onset. SARS-CoV-2-specific CD4^+^ T cells and CD8^+^ T cells declined with a half-life of 3–5 months. By studying antibody, memory B cell, CD4^+^ T cell, and CD8^+^ T cell memory to SARS-CoV-2 in an integrated manner, we observed that each component of SARS-CoV-2 immune memory exhibited distinct kinetics.

Coronavirus disease 2019 (COVID-19), caused by the novel severe acute respiratory syndrome coronavirus 2 (SARS-CoV-2), is a serious disease that has resulted in widespread global morbidity and mortality. Humans make SARS-CoV-2-specific antibodies, CD4^+^ T cells, and CD8^+^ T cells in response to SARS-CoV-2 infection (1–4). Studies of acute and convalescent COVID-19 patients have observed that T cell responses are associated with reduced disease (5–7), suggesting that SARS-CoV-2-specific CD4^+^ T cell and CD8^+^ T cell responses may be important for control and resolution of primary SARS-CoV-2 infection. Ineffective innate immunity has been strongly associated with a lack of control of primary SARS-CoV-2 infection and a high risk of fatal COVID-19 (8–12), accompanied by innate cell immunopathology (13–18). Neutralizing antibodies have generally not correlated with lessened COVID-19 disease severity (5, 19, 20), which was also observed for Middle Eastern respiratory syndrome (MERS), caused by MERS-CoV (21). Instead, neutralizing antibodies are associated with protective immunity against secondary infection with SARS-CoV-2 or SARS-CoV in non-human primates (3, 22–25). Passive transfer of neutralizing antibodies in advance of infection (mimicking preexisting conditions upon secondary exposure) effectively limits upper respiratory tract (URT) infection, lower respiratory tract (lung) infection, and symptomatic disease in animal models (26–28). Passive transfer of neutralizing antibodies provided after initiation of infection in humans have had more limited effects on COVID-19 (29, 30), consistent with a substantial role for T cells in control and clearance of an ongoing SARS-CoV-2 infection. Thus, studying antibody, memory B cell, CD4^+^ T cell, and CD8^+^ T cell memory to SARS-CoV-2 in an integrated manner is likely important for understanding the durability of protective immunity against COVID-19 generated by primary SARS-CoV-2 infection (1, 19, 31).

While sterilizing immunity against viruses can only be accomplished by high-titer neutralizing antibodies, successful protection against clinical disease or death can be accomplished by several other immune memory scenarios. Possible mechanisms of immunological protection can vary based on the relative kinetics of the immune memory responses and infection. For example, clinical hepatitis after hepatitis B virus (HBV) infection is prevented by vaccine-elicited immune memory even in the absence of circulating antibodies, because of the relatively slow course of HBV disease (32, 33). The relatively slow course of severe COVID-19 in humans (median 19 days post-symptom onset (PSO) for fatal cases (34)) suggests that protective immunity against symptomatic or severe secondary COVID-19 may involve memory compartments such as circulating memory T cells and memory B cells (which can take several days to reactivate and generate recall T cell responses and/or anamnestic antibody responses) (19, 21, 31).

Immune memory, from either primary infection or immunization, is the source of protective immunity from a subsequent infection (35–37). Thus, COVID-19 vaccine development relies on immunological memory (1, 3). Despite intensive study, the kinetics, duration, and evolution of immune memory in humans to infection or immunization are not in general predictable based on the initial effector phase, and immune responses at short time points after resolution of infection are not very predictive of long-term memory (38–40). Thus, assessing responses over an interval of six months or more is usually required to ascertain the durability of immune memory.

A thorough understanding of immune memory to SARS-CoV-2 requires evaluation of its various components, including B cells, CD8^+^ T cells, and CD4^+^ T cells, as these different cell types may have immune memory kinetics relatively independent of each other. Understanding the complexities of immune memory to SARS-CoV-2 is key to gain insights into the likelihood of durability of protective immunity against re-infection with SARS-CoV-2 and secondary COVID-19 disease. In the current study, we assessed immune memory of all three branches of adaptive immunity (CD4^+^ T cell, CD8^+^ T cell, and humoral immunity) in a predominantly cross-sectional study of 188 recovered COVID-19 cases, extending up to eight months post-infection. The findings have implications for immunity against secondary COVID-19, and thus the potential future course of the pandemic (41, 42).

## COVID-19 cohort

188 individuals with COVID-19 were recruited for this study. Subjects (80 male, 108 female) represented a range of asymptomatic, mild, moderate, and severe COVID-19 cases (Table 1), and were recruited from multiple sites throughout the United States. The majority of subjects were from California or New York. Most subjects had a “mild” case of COVID-19, not requiring hospitalization. 93% of subjects were never hospitalized for COVID-19; 7% of subjects were hospitalized, some of whom required intensive care unit (ICU) care (Table 1). This case severity distribution was consistent with the general distribution of symptomatic disease severity among COVID-19 cases in the USA. The study primarily consisted of symptomatic disease cases (97%, Table 1), due to the nature of the study recruitment design. Subject ages ranged from 19 to 81 years old (Table 1). Most subjects provided a blood sample at a single time point, between 6 days post-symptom onset (PSO) and 240 days PSO (Table 1), with 43 samples at ≥ 6 months PSO (178 days or longer). Additionally, 51 subjects in the study provided longitudinal blood samples over a duration of several months (2–4 time points; Table 1), allowing for longitudinal assessment of immune memory in a subset of the cohort.

## SARS-CoV-2 circulating antibodies over time

**Fig. 1.**
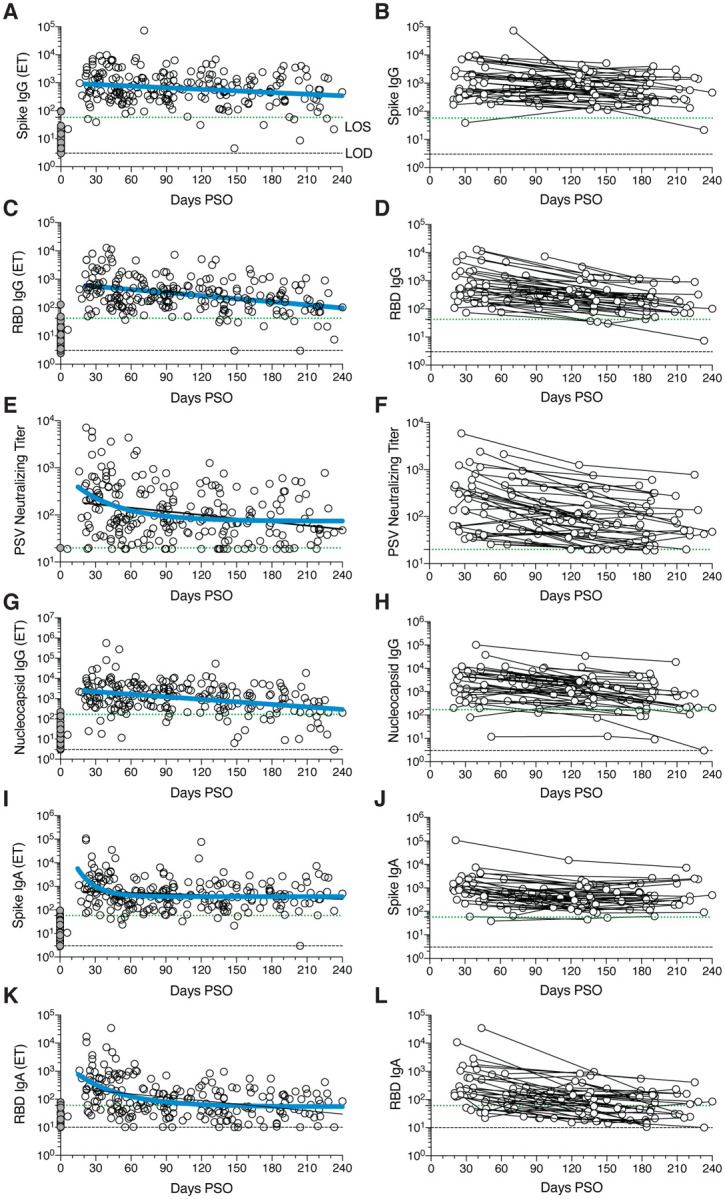
Circulating antibodies to SARS-CoV-2 over time. **(A)** Cross-sectional Spike IgG from COVID-19 subject plasma samples (n=228). Continuous decay preferred model for best fit curve, *t*_1/2_ = 140 days, 95% CI: 89–325 days. R = −0.23, p=0.0006. **(B)** Longitudinal Spike IgG (n=51), average *t*_1/2_ = 103 days, 95% CI: 65–235 days **(C)** Cross-sectional RBD IgG. Continuous decay preferred model for best fit curve, *t*_1/2_ = 83 days, 95% CI: 62 to 126 days. R = −0.36, p<0.0001. **(D)** Longitudinal RBD IgG, average *t*_1/2_ = 69 days, 95% CI: 58–87 days **(E)** Cross-sectional SARS-CoV-2 PSV neutralizing titers. One-phase decay (blue line) preferred model for best fit curve, initial *t*_1/2_ = 27 days, 95% CI 11–157d. R = −0.32. Continuous decay fit line shown as black line. **(F)** Longitudinal PSV neutralizing titers of SARS-CoV-2 infected subjects, average *t*_1/2_ = 90 days, 95% CI: 70–125 days. **(G)** Cross-sectional Nucleocapsid IgG. Continuous decay preferred model for best fit curve, *t*_1/2_ = 68 days, 95% CI: 50–106 days. R = −0.34, p<0.0001. **(H)** Longitudinal Nucleocapsid IgG, average *t*_1/2_ = 68 days, 95% CI: 55–90 days. **(I)** Cross-sectional Spike IgA titers. One-phase decay (blue line) preferred model for best fit curve, initial *t*_1/2_ = 11 days, 95% CI 5–25d. R = −0.30. Continuous decay fit shown as black line. **(J)** Longitudinal Spike IgA, *t*_1/2_ = 210 days, 95% CI 126–627 days. **(K)** Cross-sectional RBD IgA. One-phase decay (blue line) preferred model for best fit curve, initial *t*_1/2_ = 27 days, 95% CI: 15–59 days. R = −0.45. Continuous decay line fit shown in black. **(L)** Longitudinal RBD IgA, average *t*_1/2_ = 74 days, 95% CI: 56–107 days. For cross-sectional analyses, SARS-CoV-2 infected subjects (white circles, n=238) and unexposed subjects (gray circles, n=51). For longitudinal samples, SARS-CoV-2 subjects (n=51). The dotted black line indicates limit of detection (LOD). The dotted green line indicates limit of sensitivity (LOS) above uninfected controls. Unexposed = gray, COVID subjects = white. Log data analyzed in all cases. Thick blue line represents best fit curve. When two fit curves are shown, the thin black line represents the alternative fit curve.

The vast majority of SARS-CoV-2 infected individuals seroconvert, at least for a duration of months (1, 2, 4, 43–45). Seroconversion rates range from 91–99% in large studies (44, 45). Durability assessments of circulating antibody titers in Figure 1 were based on data ≥ 20 days PSO, with the plot of the best fitting curve fit model shown in blue (see Methods). SARS-CoV-2 Spike immunoglobulin G (IgG) endpoint ELISA titers in plasma were measured for all subjects of this cohort (Fig. 1A–B). Spike receptor binding domain (RBD) IgG was also measured (Fig. 1C–D), as RBD is the target of most neutralizing antibodies against SARS-CoV-2 (4, 27, 46, 47). SARS-CoV-2 pseudovirus (PSV) neutralizing antibody titers were measured in all subjects (Fig. 1E–F). Nucleocapsid (N) IgG endpoint ELISA titers were also measured for all subjects (Fig. 1G–H), as Nucleocapsid is a common antigen in commercial SARS-CoV-2 serological test kits.

SARS-CoV-2 Spike IgG titers were relatively stable from 20–240 days PSO, when assessing all COVID-19 subjects by cross-sectional analysis (half-life *t*_1/2_ = 140 days, Fig. 1A). Spike IgG titers were heterogenous among subjects (range 5 to 73,071; 575 median), as has been widely observed (45, 47). This gave a wide confidence interval for the Spike IgG *t*_1/2_ (95% CI: 89 to 325 days). While the antibody responses may have more complex underlying decay kinetics, the best fit curve was a continuous decay, likely related to heterogeneity between individuals. SARS-CoV-2 Nucleocapsid IgG kinetics were similar to Spike IgG over 8 months (*t*_1/2_ 68 days, 95% CI: 50–106 days. Fig. 1G). As a complementary approach, using paired samples from the subset of subjects who donated at two or more time points, the calculated Spike IgG titer average *t*_1/2_ was 103 days, (95% CI: 66–235 days, Fig. 1B) and the Nucleocapsid IgG titer average *t*_1/2_ was 68 days, (95% CI: 55–90 days, Fig. 1H). The percentage of subjects seropositive for Spike IgG at 1 month PSO (20–50 days) was 98% (54/55). The percentage of subjects seropositive for Spike IgG at 6 to 8 months PSO (≥ 178 days) was 90% (36/40).

Cross-sectional analysis of SARS-CoV-2 RBD IgG titers from 20–240 days PSO gave an estimated *t*_1/2_ of 83 days (95% CI: 62–126 days, Fig. 1C). As a complementary approach, we again used paired samples, which gave an average *t*_1/2_ of 69 days (95% CI: 58–87 days, Fig. 1D). The percentage of subjects seropositive for RBD IgG at 6 to 8 months PSO was 88% (35/40). Thus, RBD IgG titer maintenance largely matched that of Spike IgG. SARS-CoV-2 PSV neutralization titers in the full cohort largely matched the results of SARS-CoV-2 RBD IgG ELISA binding titers (Fig.1E–F). A one-phase decay model was the best fit (P=0.015, F test. Initial decay *t*_1/2_ 27 days, followed by an extended plateau phase. Fig. 1E), while a continuous decay fit gave an estimated *t*_1/2_ of 114 days (Fig. 1E, black line). Paired timepoints analysis of the PSV neutralization titers gave an estimated *t*_1/2_ of 90 days, (95% CI: 70–125 days, Fig. 1F). The percentage of subjects seropositive for SARS-CoV-2 neutralizing antibodies (titer ≥ 20) at 6 to 8 months PSO was 90% (36/40). Notably, even low levels of circulating neutralizing antibody titers (≥ 1:20) were associated with a substantial degree of protection against COVID-19 in non-human primates (24, 48). Thus, modest levels of circulating SARS-CoV-2 neutralizing antibodies are of biological interest in humans.

SARS-CoV-2 Spike IgA (Fig. 1I–J) and RBD IgA (Fig.1K–L) titers were also assessed. Paired timepoints analysis of Spike IgA titers yielded an estimated *t*_1/2_ of 210 days (95% CI 126–703 days, Fig. 1J). Cross-sectional analysis of Spike IgA fit a short one-phase decay model with an extended plateau phase (initial *t*_1/2_ of 14 days, Fig. 1I). Circulating RBD IgA had an estimated initial *t*_1/2_ of 27 days, decaying by ~90 days in most COVID-19 cases to levels indistinguishable from uninfected controls (Fig. 1K), consistent with observations 3 months PSO (44, 49). By paired sample analysis, long-lasting RBD IgA was made in some subjects, but often near the limit of sensitivity (LOS) (Fig. 1L).

## SARS-CoV-2 memory B cells

**Fig. 2.**
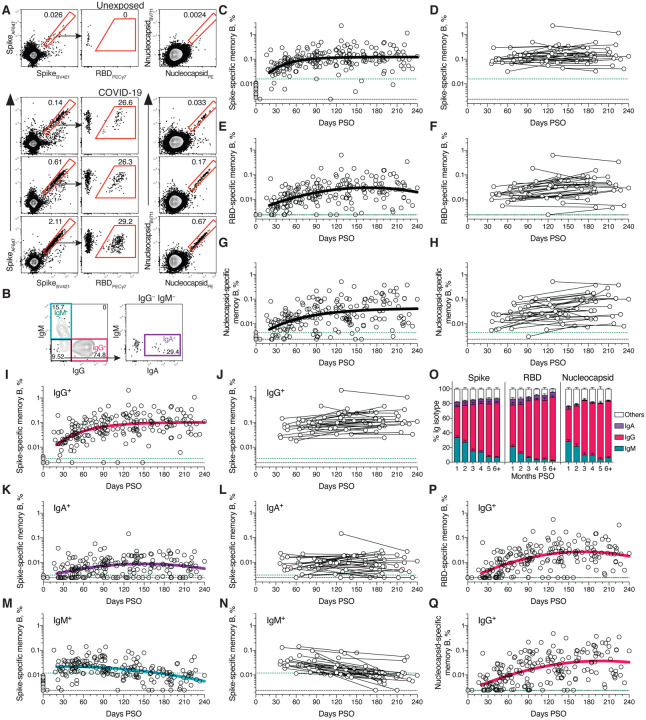
Kinetics of SARS-CoV-2 memory B cell responses. (**A**) Example flow cytometry plots showing staining patterns of SARS-CoV-2 antigen probes on memory B cells (See Fig. S1 for gating). One unexposed donor and three convalescent COVID-19 subjects are shown. Numbers indicate percentages. (**B**) Gating strategies to define IgM^+^, IgG^+^, or IgA^+^ SARS-CoV-2 Spike-specific memory B cells. The same gating strategies were used for RBD- or Nucleocapsid-specific B cells. (**C**) Cross-sectional analysis of frequency (% of CD19^+^ CD20^+^ B cells) of SARS-CoV-2 S-specific total (IgG^+^, IgM^+^, or IgA^+^) memory B cells. Pseudo-first order kinetic model for best fit curve (R = 0.38). (**D**) Longitudinal analysis of SARS-CoV-2 Spike-specific memory B cells. (**E**) Cross-sectional analysis of SARS-CoV-2 RBD-specific total (IgG^+^, IgM^+^, or IgA^+^) memory B cells. Second order polynomial model for best fit curve (R = 0.46). (**F**) Longitudinal analysis of SARS-CoV-2 RBD-specific memory B cells. (**G**) Cross-sectional analysis of SARS-CoV-2 Nucleocapsid-specific total (IgG^+^, IgM^+^, or IgA^+^) memory B cells. Pseudo-first order kinetic model for best fit curve (R = 0.44). (**H**) Longitudinal analysis of IgG^+^ SARS-CoV-2 Nucleocapsid-specific memory B cells. (**I**) Cross-sectional analysis of SARS-CoV-2 Spike-specific IgG^+^ memory B cells. Pseudo-first order kinetic model for best fit curve (R = 0.49). (**J**) Longitudinal analysis of SARS-CoV-2 Spike-specific IgG^+^ memory B cells. (**K**) Cross-sectional analysis of SARS-CoV-2 Spike-specific IgA^+^ memory B cells. Second order polynomial model for best fit curve (|R| = 0.32). (**L**) Longitudinal analysis of SARS-CoV-2 Spike-specific IgA^+^ memory B cells. (**M**) Cross-sectional analysis of SARS-CoV-2 Spike-specific IgM^+^ memory B cells. Second order polynomial model for best fit curve (|R| = 0.41). (**N**) Longitudinal analysis of SARS-CoV-2 Spike-specific IgM^+^ memory B cells. (**O**) Fraction of SARS-CoV-2 antigen-specific memory B cells that belong to indicated Ig isotypes at 1–8 months PSO. Mean ± SEM. (**P**) Cross-sectional analysis of SARS-CoV-2 RBD-specific IgG^+^ memory B cells. Second order polynomial model for best fit curve (|R| = 0.51). (**Q**) Cross-sectional analysis of SARS-CoV-2 Nucleocapsid-specific IgG^+^ memory B cells. Second order polynomial model for best fit curve (|R| = 0.51). n = 20 unexposed subjects (gray circles) and n = 160 COVID-19 subjects (n = 197 data points, white circles) for cross-sectional analysis. n = 36 COVID-19 subjects (n = 73 data points, white circles) for longitudinal analysis. The dotted black line indicates limit of detection (LOD). The dotted green line indicates limit of sensitivity (LOS).

To identify SARS-CoV-2-specific memory B cells, fluorescently labeled multimerized probes were used to detect B cells specific to Spike, RBD, and Nucleocapsid (Fig 2A, Fig. S1). Antigen-binding memory B cells (defined as IgD^−^ and/or CD27^+^) were further distinguished according to surface Ig isotypes: IgM, IgG or IgA (Fig. 2B, Fig. S1).

Cross-sectional analysis of COVID-19 subjects revealed that frequencies of SARS-CoV-2 Spike-specific memory B cells increased over the first ~120 days PSO and then plateaued (pseudo-first order model for best fit curve, R = 0.38. Better fit than second order polynomial model by Akaike’s Information Criterion. Fig 2C, Fig. S2A). Spike-specific memory B cell frequencies increased from the first time-point (36–163 days) to the second time-point (111–240 days) in paired samples from 24 of 36 longitudinally tracked donors (Fig 2D). Spike-specific memory B cells in SARS-CoV-2-unexposed subjects were rare (median 0.0078%. Fig 2A, 2C).

RBD-specific memory B cells displayed similar kinetics to Spike-specific memory B cells. RBD-specific memory B cells were undetectable in SARS-CoV-2 unexposed subjects (Fig. 2E. Fig. S2C), as expected. RBD-specific memory B cells appeared as early as 16 days PSO, and the frequency steadily increased in the following 4–5 months (Fig. 2E. Fig. S2B–C). 29 of 36 longitudinally tracked individuals had higher frequencies of RBD-specific memory B cells at the later time point (Fig. 2F), again showing an increase in SARS-CoV-2 specific memory B cells several months post-infection. ~10–30% of Spike-specific memory B cells from SARS-CoV-2 convalescent donors were specific for the RBD domain (Fig. 2A, Fig. S2B).

SARS-CoV-2 Nucleocapsid-specific memory B cells were also detected after SARS-CoV-2 infection (Fig. 2A). Similar to Spike- and RBD-specific memory B cells, Nucleocapsid-specific memory B cell frequency steadily increased during the first ~4–5 months PSO (Fig. 2G, 2H, Fig. S2D). Antibody affinity maturation could potentially explain the increased frequencies of SARS-CoV-2-specific memory B cells detected by the antigen probes. However, geometric mean fluorescent intensity (MFI) of probe binding was stable over time (Fig. S2I–J), not supporting an affinity maturation explanation for the increased memory B cell frequencies.

Representation of Ig isotypes among the SARS-CoV-2 Spike-specific memory B cell population shifted with time (Fig. 2I–2O). During the earliest phase of memory (20–60 days PSO), IgM^+^ and IgG^+^ isotypes were similarly represented (Fig. 2O), but IgM^+^ memory B cells then declined (Fig. 2M–O), and IgG^+^ Spike-specific memory B cells then dominated by 6 months PSO (Fig. 2O). IgA^+^ Spike-specific memory B cells were detected as a small fraction of the total Spike-specific memory B cells (~5%, Fig. 2O). IgG^+^ Spike-specific memory B cell frequency increased while IgA^+^ was low and stable over the 8 months period (Fig. 2I–2L). Similar patterns of increasing IgG^+^ memory, short-lived IgM^+^ memory, and stable IgA^+^ memory were observed for RBD- and Nucleocapsid-specific memory B cells over the 8 months period (Fig. 2O–2Q, Fig. S2E–S2H).

There is limited knowledge of memory B cell kinetics following primary acute viral infection in humans. A recently published SARS-CoV-2 study found RBD-specific memory B cells out to ~90 days PSO, with increasing frequencies (and a low frequency of IgA^+^ cells) (50), consistent with observations reported here. For other acute infectious diseases, we are not currently aware of other cross-sectional or longitudinal analyses of antigen-specific memory B cells by flow cytometry covering a 6+ month window after infection, except for four individuals with Ebola (51) and two individuals studied after yellow fever virus immunization (52) (we exclude influenza vaccines for comparison here, because people have numerous exposures and complex immune history to influenza). In the yellow fever study, short-lived IgM^+^ memory and longer-lasting isotype-switched memory B cells were observed in the two individuals. Overall, based on the observations here, development of B cell memory to SARS-CoV-2 was robust, and is likely long-lasting.

## SARS-CoV-2 memory CD8^+^ T cells

**Fig. 3.**
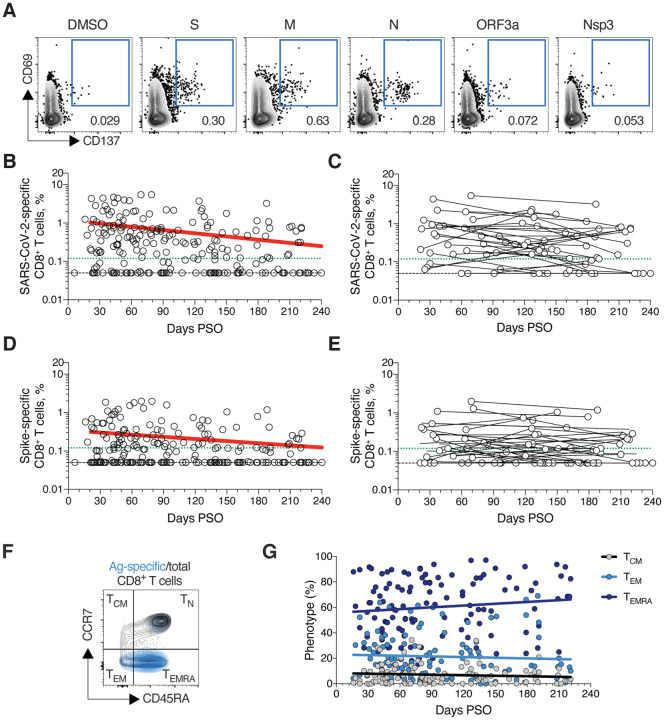
SARS-CoV-2 circulating memory CD8^+^ T cells. **(A)** Representative flow cytometry plots of SARS-CoV-2-specific CD8^+^ T cells (CD69^+^ CD137^+^, See Fig. S3 for gating) after overnight stimulation with S, N, M, ORF3a, or nsp3 peptide pools, compared to negative control (DMSO). **(B)** Cross-sectional analysis of frequency (% of CD8^+^ T cells) of total SARS-CoV-2-specific CD8^+^ T cells. Continuous decay preferred fit model, t_1/2_ = 125 days. R = −0.24, p = 0.0003. **(C)** Longitudinal analysis of total SARS-CoV-2-specific CD8^+^ T cells in paired samples. **(D)** Cross-sectional analysis of Spike-specific CD8^+^ T cells. Linear decay preferred model, t_1/2_ = 225 days. R = −0.18, p = 0.007. **(E)** Longitudinal analysis of Spike-specific CD8^+^ T cells in paired samples. **(F)** Distribution of central memory (T_CM_), effector memory (T_EM_), and terminally differentiated effector memory cells (T_EMRA_) among total SARS-CoV-2-specific CD8^+^ T cells. n = 169 COVID-19 subjects (n = 215 data points, white circles) for cross-sectional analysis. n = 37 COVID-19 subjects (n = 83 data points, white circles) for longitudinal analysis. The dotted black line indicates limit of detection (LOD). The dotted green line indicates limit of sensitivity (LOS).

SARS-CoV-2 memory CD8^+^ T cells were measured in 169 COVID-19 subjects using a series of 23 peptide pools covering the entirety of the SARS-CoV-2 ORFeome (2, 5). The most commonly recognized ORFs were Spike, Membrane (M), Nucleocapsid, and ORF3a (CD69^+^ CD137^+^, Fig. 3A and Fig. S3A–B), consistent with our previous study (2). The percentage of subjects with detectable circulating SARS-CoV-2 memory CD8^+^ T cells at 1 month PSO (20–50 days) was 70% (40/57, Fig. 3B). The proportion of subjects positive for SARS-CoV-2 memory CD8^+^ T cells at ≥ 6 months PSO was 50% (18/36). This could potentially underestimate CD8^+^ T cell memory, as 15-mers can be suboptimal for detection of some antigen-specific CD8^+^ T cells (53); however, pools of predicted SARS-CoV-2 class I epitope of optimal size also detected virus-specific CD8^+^ T cells in ~70% of individuals 1–2 months PSO, indicating consistency between the two experimental approaches (2).

SARS-CoV-2 memory CD8^+^ T cells declined with an apparent *t*_1/2_ of 125 days in the full cohort (Fig. 3B) and *t*_1/2_ 190 days among 29 paired samples (Fig. 3C). Spike-specific memory CD8^+^ T cells exhibited similar kinetics to the overall SARS-CoV-2-specific memory CD8^+^ T cells (*t*_1/2_ 225 days for the full cohort and 185 days among paired samples, Fig. 3D–E, respectively). Phenotypic markers indicated that the majority of SARS-CoV-2-specific memory CD8^+^ T cells were terminally differentiated effector memory cells (T_EMRA_) (54), with small populations of central memory (T_CM_) and effector memory (T_EM_) (Fig. 3F). In the context of influenza, CD8^+^ T_EMRA_ cells were associated with protection against severe disease in humans (55). The memory CD8^+^ T cell half-lives observed here were comparable to the 123 days *t*_1/2_ observed for memory CD8^+^ T cells after yellow fever immunization (56). Thus, the kinetics of circulating SARS-CoV-2-specific CD8^+^ T cell were consistent with what has been reported for another virus that causes acute infections in humans.

## SARS-CoV-2 memory CD4^+^ T cells

**Fig. 4.**
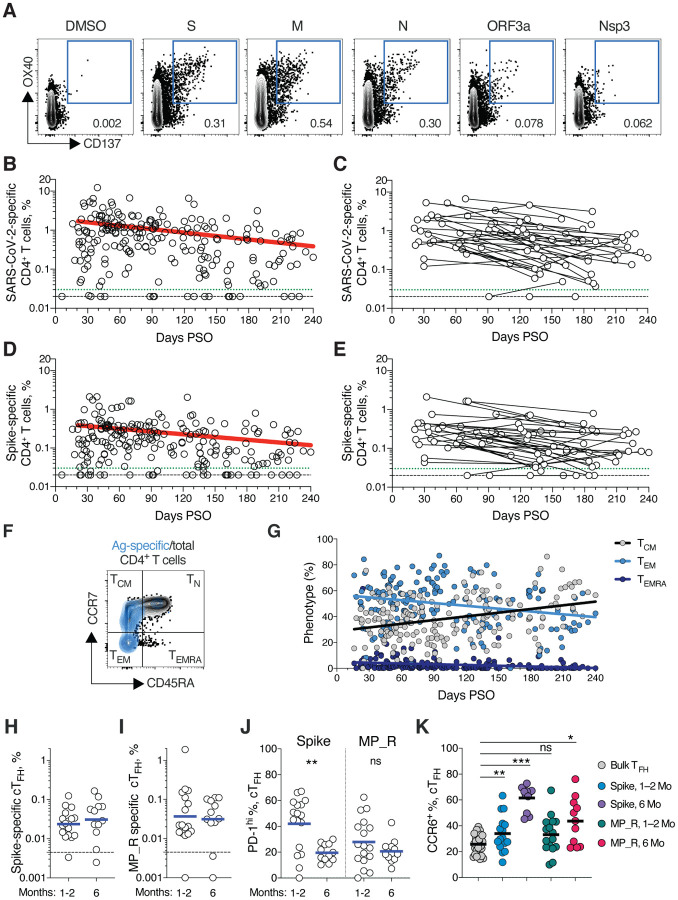
SARS-CoV-2 circulating memory CD4^+^ T cells. **(A)** Representative flow cytometry plots of SARS-CoV-2-specific CD4^+^ T cells (CD137^+^ OX40^+^, See Fig S4 for gating) after overnight stimulation with S, N, M, ORF3a, or nsp3 peptide pools, compared to negative control (DMSO). **(B)** Cross-sectional analysis of frequency (% of CD4^+^ T cells) of total SARS-CoV-2-specific CD4^+^ T cells. Continuous decay preferred fit model, *t*_1/2_ = 94 days. R = −0.29, p<0.0001. **(C)** Longitudinal analysis of total SARS-CoV-2-specific CD4^+^ T cells in paired samples from the same subjects. **(D)** Cross-sectional analysis of Spike-specific CD4^+^ T cells. Linear decay preferred model, t_1/2_ = 139 days. R = −0.26, p<0.0001. **(E)** Longitudinal analysis of Spike-specific CD4^+^ T cells in paired samples from the same subjects. **(F, G)** Distribution of central memory (T_CM_), effector memory (T_EM_), and terminally differentiated effector memory cells (T_EMRA_) among total SARS-CoV-2-specific CD4^+^ T cells. (**H, I**) Quantitation of SARS-CoV-2-specific circulating T follicular helper (cT_FH_) cells (surface CD40L^+^ OX40^+^, as % of CD4^+^ T cells. See Fig S5 for gating) after overnight stimulation with **(H)** Spike (S) or **(I)** MP_R peptide pools. (**J**) PD-1^hi^ SARS-CoV-2-specific T_FH_ at 1–2 months (mo) and 6 mo PSO. (**K**) CCR6^+^ SARS-CoV-2-specific cT_FH_ in comparison to bulk cT_FH_ cells in blood. For (**A-E**), n = 169 COVID-19 subjects (n = 215 data points, white circles) for cross-sectional analysis, n = 37 COVID-19 subjects (n = 83 data points, white circles) for longitudinal analysis. The dotted black line indicates limit of detection (LOD). The dotted green line indicates limit of sensitivity (LOS). For **(HJ**), n = 29 COVID-19 subject samples (white circles), n = 17 COVID-19 subjects at 1–2 mo, n = 12 COVID-19 subjects at 6 mo. The dotted black line indicates limit of detection (LOD). Statistics by (**J**) Mann-Whitney U test and (**K**) Wilcoxon signed-rank test. * p<0.05, **p<0.01, *** p<0.001.

SARS-CoV-2 memory CD4^+^ T cells were identified in 169 subjects using the same series of 23 peptide pools covering the SARS-CoV-2 ORFeome (2, 5). The most commonly recognized ORFs were Spike, M, Nucleocapsid, ORF3a, and nsp3 (CD137^+^ OX40^+^, Fig. 4A and Fig. S4A–B), consistent with our previous study (2). Circulating SARS-CoV-2 memory CD4^+^ T cell responses were quite robust (Fig. 4B); 42% (24/57) of COVID-19 cases at 1 month PSO had > 1.0% SARS-CoV-2-specific CD4^+^ T cells. SARS-CoV-2 memory CD4^+^ T cells declined with an apparent *t*_1/2_ of 94 days in the full cohort (Fig. 4B) and *t*_1/2_ 64 days among 36 paired samples (Fig. 4C). The percentage of subjects with detectable circulating SARS-CoV-2 memory CD4^+^ T cells at 1 month PSO (20–50 days) was 93% (53/57, Fig. 4B). The proportion of subjects positive for SARS-CoV-2 memory CD4^+^ T cells at ≥ 6 months PSO was 92% (33/36).

Spike-specific and M-specific memory CD4^+^ T cells exhibited similar kinetics to the overall SARS-CoV-2-specific memory CD4^+^ T cells (whole cohort *t*_1/2_ 139 days and 153 days, respectively. Fig. 4D–E, and Fig. S4D). A plurality of the SARS-CoV-2 memory CD4^+^ T cells present at ≥ 6 months PSO had a T_CM_ phenotype (Fig. 4F).

T follicular helpers (T_FH_) are the specialized subset of CD4^+^ T cells required for B cell help (57), and are therefore critical for the generation of neutralizing antibodies and long-lived humoral immunity in most contexts. Thus, we examined circulating T_FH_ (cT_FH_) memory CD4^+^ T cells, with particular interest in Spike-specific memory cT_FH_ cells due to the importance of antibody responses against Spike. Memory cT_FH_ cells specific for predicted epitopes across the remainder of the SARS-CoV-2 genome were also measured, using the MP_R megapool. Memory cT_FH_ cells specific for SARS-CoV-2 Spike and MP_R were detected in the majority of COVID-19 cases at early time points (16/17. Fig. 4H–I, and Fig. S5A–D). cT_FH_ memory appeared to be stable, with almost all subjects positive for Spike and MP_R memory cT_FH_ cells at 6 months PSO (11/12 & 10/12, respectively. Fig. 4H–I). Recently activated cT_FH_ cells are PD-1^hi^ (57). Consistent with conversion to resting memory cT_FH_ cells, the percentage of PD-1^hi^ SARS-CoV-2-specific memory cT_FH_ dropped over time (Fig. 4J). CCR6^+^ SARS-CoV-2-specific cT_FH_ cells have been associated with reduced COVID-19 disease severity (5) and have been reported to be a major fraction of Spike-specific cT_FH_ cells in some studies (5, 50, 58). Here we confirmed that a significant fraction of both Spike-specific and MP_R memory cT_FH_ cells were CCR6^+^. We also observed increases in CCR6^+^ cT_FH_ memory over time (p=0.001 and p=0.014 at > 6 months PSO compared to bulk cT_FH_. Fig. 4K). Overall, substantial cT_FH_ memory was observed after SARS-CoV-2 infection, with durability ≥ 6 months PSO.

## Immune memory relationships

**Fig. 5.**
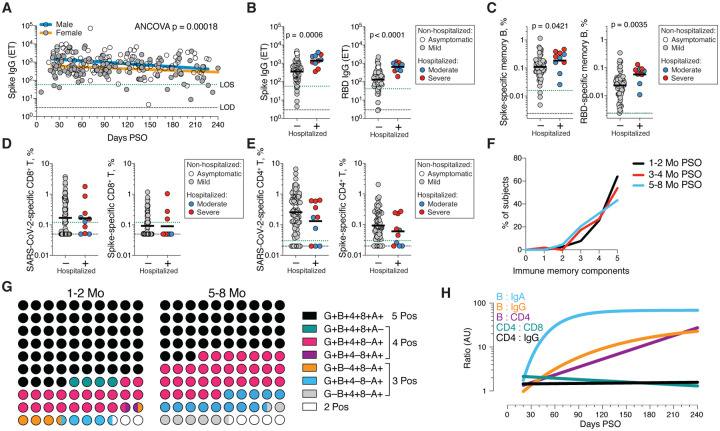
Immune memory relationships. **(A)** Relationship between gender and Spike IgG titers over time. Males: Linear decay preferred model, *t*_1/2_ = 110 days, 95% CI: 65–349 days, R = −0.27, p = 0.0046. Females: linear decay preferred model, *t*_1/2_ = 159 days, 95% CI 88–846 days, R = −0.22, p = 0.016. ANCOVA p = 0.00018. Test for homogeneity of regressions F = 1.51, p = 0.22. **(B-E)** Immune memory at 120+ days PSO in COVID-19 non-hospitalized and hospitalized subjects. Symbol colors represent peak disease severity (white: asymptomatic, gray: mild, blue: moderate, red: severe.) For subjects with multiple sample timepoints, only the final timepoint was used for these analyses. **(B)** Spike-specific IgG (left) and RBD-specific IgG (right) binding titers. n = 64 (non-hospitalized), n = 10 (hospitalized). Mann-Whitney U tests. (**C**) Frequency memory B cells specific to Spike (left) and RBD (right) at 120+ days PSO. n = 66 (non-hospitalized), n = 10 (hospitalized). Mann-Whitney U tests. (**D**) Frequency total SARS-CoV-2-specific CD8^+^ T cells (left) and Spike-specific CD8^+^ T cells (right). p = 0.72 for total SARS-2-CoV-specific, p = 0.60 for Spike-specific by Mann-Whitney U tests. n = 72 (non-hospitalized), n = 10 (hospitalized). (**E**) Frequency total SARS-CoV-2-specific CD4^+^ T cells (left) and Spike-specific CD4^+^ T cells (right). p = 0.23 for total SARS-CoV-2-specific, p = 0.24 for Spike-specific by Mann-Whitney U tests (**F**) Immune memory to SARS-CoV-2 during the early phase (1–2 mo, black line), medium phase (3–4 mo, red line), or late phase (5–8 mo, blue line). For each individual, a score of 1 was assigned for each response above LOS for RBD IgG, Spike IgA, RBD-specific memory B cells, SARS-CoV-2 specific CD4^+^ T cells, and SARS-CoV-2-specific CD8^+^ T cells, giving a maximum total of 5 components of SARS-CoV-2 immune memory. Only COVID-19 convalescent subjects with all five immunological parameters tested were included in the analysis. n = 78 (1–2 mo), n = 52 (3–4 mo), n = 44 (5–8 mo). **(G)** Percentage dot plots showing frequencies (normalized to 100%) of subjects with indicated immune memory components as described in (B) during the early (1–2 mo) or late (5–8 mo) phase. “G”, RBD-specific IgG. “B”, RBD-specific memory B cells. “4”, SARS-CoV-2 specific CD4^+^ T cells. “8”, SARS-CoV-2 specific CD8^+^ T cells. “A”, Spike-specific IgA. n = 78 (1–2 mo), n = 44 (5–8 mo). **(H)** Relationships between immune memory compartments in COVID-19 subjects over time, as ratios (full curves and data shown in Fig. S10B–F). AU = arbitrary units, scaled from Fig. S10B–F. “B/IgA”, RBD-specific memory B cell ratio to Spike IgA antibodies. “B/IgG”, RBD-specific memory B cell ratio to RBD IgG antibodies. “B/CD4”, RBD-specific memory B cell ratio to SARS-CoV-2-specific CD4^+^ T cells. “CD4/CD8”, SARS-CoV-2-specific CD4^+^ T cells ratio to SARS-CoV-2-specific CD8^+^ T cells. “CD4/IgG”, SARS-CoV-2-specific CD4^+^ T cells ratio to RBD IgG antibodies.

Immune memory to SARS-CoV-2 were considered, including relationships between the compartments of immune memory. Males had higher Spike IgG (ANCOVA p=0.00018, Fig. 5A) and RBD and Nucleocapsid IgG (ANCOVA p=0.00077 & p=0.018, Fig. S6A–B), consistent with other studies (46, 47). Higher Spike IgG was also observed in males when only non-hospitalized cases were considered (ANCOVA p=0.00025, Fig. S6C). In contrast, no differences were observed in IgA or PSV neutralization titers (Fig. S6D–F), and no differences were detected in SARS-CoV-2 memory B cell, memory CD8^+^ T cell, or memory CD4^+^ T cell frequencies between males and females (Fig. S6G–K).

Immune memory was examined for associations between magnitude of memory and COVID-19 disease severity. The number of previously hospitalized COVID-19 cases (n=13) limited analysis options. However, the cases were well distributed between males and females (Table 1), data from large numbers of non-hospitalized cases were available for comparison, and the analyses in Figures 1–4 demonstrated that immune memory was relatively stable over the time window analyzed. Therefore, we could simplify the disease severity analysis by grouping all samples from 120+ days PSO (also limiting data to a single sample per subject [Fig. S7–9]; most of the previously hospitalized subjects were sampled at two timepoints. Fig. S7A) and then comparing non-hospitalized and hospitalized subjects. Spike and RBD IgG titers in hospitalized cases were higher than non-hospitalized cases (Fig. 5B), consistent with other studies (46, 47). Spike and RBD-specific memory B cell frequencies were also higher in hospitalized cases (~1.7-fold and ~ 2.5-fold, respectively. Fig. 5C, Fig S8). In contrast, memory CD8^+^ T cell frequencies were not higher in hospitalized cases compared to non-hospitalized cases (Fig. 5D, Fig. S9) and memory CD4^+^ T cell frequencies trended lower in hospitalized cases compared to non-hospitalized cases (Fig. 5E, Fig. S9). Therefore, while conclusions are limited by the number of hospitalized subjects, increased Spike IgG titers was consistent across three independent studies, and increased memory B cells among hospitalized cases were observed here (not measured in other studies), indicating that both compartments of long-term humoral immunity to SARS-CoV-2 are higher in individuals who experienced a more severe COVID-19 disease course. T cell memory did not follow the same pattern, consistent with indications that hospitalized cases of COVID-19 can be associated with poorer T cell responses in the acute phase (5, 59). Additionally, these data show that, while gender and COVID-19 disease severity contribute to differences in immune memory to SARS-CoV-2, neither factor could account for the majority of the heterogeneity in immune memory to this virus.

Very few published data sets compare antigen-specific antibody, B cell, CD8^+^ T cell, and CD4^+^ T cell memory to an acute viral infection in the same individuals. We therefore made use of this combined data set to examine interrelationships between compartments of immune memory. We focused on RBD IgG, RBD memory B cells, Spike IgA, total SARS-CoV-2-specific CD8^+^ T cells, and total SARS-CoV-2-specific CD4^+^ T cells, due to their putative potential roles in protective immunity. The majority (64%) of COVID-19 cases were positive for all five of these immune memory compartments at 1 to 2 months PSO (Fig. 5F–G), with the incomplete responses largely reflecting individuals with no detectable CD8^+^ T cell memory and/or poor IgA responses (Fig. 5G). At 5 to 8 months after COVID-19, the proportion of individuals positive for all five of these immune memory compartments had dropped to 43%; nevertheless, 95% of individuals were still positive for at least three out of five SARS-CoV-2 immune memory responses (Fig. 5G). Immune memory at 5 to 8 months PSO represented contributions from different immune memory compartments in different individuals (Fig. 5G). Similar results were obtained if RBD IgG was replaced by neutralizing antibodies (Fig. S10A). Overall, these findings again highlight heterogeneity of immune memory, with different patterns of immune memory in different individuals.

Interrelationships between the components of memory were next examined by assessing ratios between immune memory compartments over time. The ratio of SARS-CoV-2 CD4^+^ T cell memory to SARS-CoV-2 CD8^+^ T cell memory was largely stable over time (Fig. 5H, Fig. S10B). Given that serological measurements are the simplest measurements of immune memory at a population scale, we examined how well such serological measurements may serve as surrogate markers of other components of SARS-CoV-2 immune memory over time. The relationship between circulating RBD IgG and RBD-specific memory B cells changed ~20-fold over the time range studied (R=0.60, Fig. 5H, Fig. S10C). The changing relationship between circulating Spike IgA and RBD-specific memory B cells was even larger (R=0.55, Fig. 5H, Fig. S10D). The relationship between RBD IgG and SARS-CoV-2 CD4^+^ T cell memory was relatively flat over the time range studied (Fig. 5H); however, variation spanned a ~1000-fold range (Fig. S10E). Thus, predictive power of circulating RBD IgG for assessing T cell memory was poor because of the heterogeneity between individuals (R=0.046). In sum, while heterogeneity of immune responses is a defining feature of COVID-19, immune memory to SARS-CoV-2 develops in almost all subjects, with complex relationships between the individual immune memory compartments.

## Concluding remarks

In this study, we aimed to fill gaps in our basic understanding of immune memory after COVID-19. This required simultaneous measurement of circulating antibodies, memory B cells, CD8^+^ T cells, and CD4^+^ T cells specific for SARS-CoV-2, in a group of subjects with a full range of disease, and distributed from short time points after infection out to 8 months later. By studying these multiple compartments of adaptive immunity in an integrated manner, we observed that each component of SARS-CoV-2 immune memory exhibited distinct kinetics.

The Spike IgG titers were durable, with modest declines in titers at 6 to 8 months PSO at the population level. RBD IgG and SARS-CoV-2 PSV neutralizing antibody titers were potentially similarly stable, consistent with the RBD domain of Spike being the dominant neutralizing antibody target. We collected data at two time points for most longitudinal individuals herein. It is well recognized that the magnitude of the antibody response against SARS-CoV-2 is highly heterogenous between individuals. We observed that heterogenous initial antibody responses did not collapse into a homogeneous circulating antibody memory; rather, heterogeneity is also a central feature of immune memory to this virus. For antibodies, the responses spanned a ~200-fold range. Additionally, this heterogeneity means that long-term longitudinal studies will be required to precisely define antibody kinetics to SARS-CoV-2. We are reporting the simplest statistical models that explain the data. These curve fits do not disprove more complex kinetics such as overlapping kinetics, but those models would require much denser longitudinal sampling in future studies. Nevertheless, at 5 to 8 months PSO, almost all individuals were positive for SARS-CoV-2 Spike and RBD IgG.

Notably, memory B cells specific for the Spike protein or RBD were detected in almost all COVID-19 cases, with no apparent half-life at 5 to 8 months post-infection. Other studies of RBD memory B cells are reporting similar findings (50, 60). B cell memory to some other infections has been observed to be long-lived, including 60+ years after smallpox vaccination (61), or 90+ years after infection with influenza (62). The memory T cell half-lives observed over 6+ months PSO in this cohort (~125–225 days for CD8^+^ and ~94–153 days for CD4^+^ T cells) were comparable to the 123 days *t*_1/2_ observed for memory CD8^+^ T cells after yellow fever immunization (56). SARS-CoV-2 T cell memory at 6 months has also now been reported in another study (63). Notably, the durability of a fraction of the yellow fever virus-specific memory CD8^+^ T cells possessed an estimated *t*_1/2_ of 485 days by deuterium labeling (56). Using different approaches, the long-term durability of memory CD4^+^ T cells to smallpox, over a period of many years, was an estimated *t*_1/2_ of ~10 years (61, 64), which is also consistent with recent detection of SARS-CoV-T cells 17 years after the initial infection (65). These data suggest that T cell memory might reach a more stable plateau, or slower decay phase, beyond the first 8 months post-infection.

While immune memory is the source of long-term protective immunity, direct conclusions about protective immunity cannot be made on the basis of quantifying SARS-CoV-2 circulating antibodies, memory B cells, CD8^+^ T cells, and CD4^+^ T cells, because mechanisms of protective immunity against SARS-CoV-2 or COVID-19 are not defined in humans. Nevertheless, some reasonable interpretations can be made. Antibodies are the only component of immune memory that can provide truly sterilizing immunity. Immunization studies in non-human primates have indicated that circulating neutralization titers of ~200 may provide sterilizing immunity against a relatively high dose URT challenge (66), and neutralizing titers of ~3,400 may provide sterilizing immunity against a very high dose URT challenge (67), although direct comparisons are not possible because the neutralizing antibody assays have not been standardized (3). Conclusions are also constrained by the limited overall amount of data on protective immunity to SARS-CoV-2.

Beyond sterilizing immunity, immune responses that confine SARS-CoV-2 to the URT and oral cavity would minimize COVID-19 disease severity to that of a ‘common cold’ or asymptomatic disease. This outcome is the primary goal of current COVID-19 vaccine clinical trials (3, 68). Such an outcome could potentially be mediated by a mixture of memory CD4^+^ T cells, memory CD8^+^ T cells, and memory B cells specific for RBD producing anamnestic neutralizing antibodies, based on mechanisms of action in mouse models of other viral infections (69–71). In human COVID-19 infections, SARS-CoV-2-specific CD4^+^ T cells and CD8^+^ T cells are associated with less COVID-19 disease severity during an ongoing SARS-CoV-2 infection (5). Rapid seroconversion was associated with significantly reduced viral loads in acute disease over 14 days (29). Both of those associations are consistent with the hypothesis that SARS-CoV-2 memory T cells and B cells would be capable of substantially limiting SARS-CoV-2 dissemination and/or cumulative viral load, resulting in reduced COVID-19 disease severity. The likelihood of such outcomes is also closely tied to the kinetics of the infection, as memory B and T cell responses can take 3–5 days to successfully respond to an infection. As noted above, given the relatively slow course of severe COVID-19 in humans, resting immune memory compartments can potentially contribute in meaningful ways to protective immunity against pneumonia or severe secondary COVID-19. The presence of sub-sterilizing neutralizing antibody titers at the time of SARS-CoV-2 exposure would blunt the size of the initial infection, and may provide an added contribution to limiting COVID-19 severity, based on observations of protective immunity for other human respiratory viral infections (37, 72–74) and observations of SARS-CoV-2 vaccines in non-human primates (48, 67, 75).

The current study has some limitations. Longitudinal data for each subject, with at least three time points per subject, would be required for more precise understanding of the kinetics of durability of SARS-CoV-2 antibodies. Nevertheless, the current cross-sectional data describe well the dynamics of SARS-CoV-2 memory B cells, CD8^+^ T cell, and CD4^+^ T cell over 8 months PSO. This study was not sufficiently powered to control for many variables simultaneously. Additionally, circulating memory was assessed here; it is possible that local URT immune memory is a minimal, moderate, or large component of immune memory after a primary infection with SARS-CoV-2. This remains to be determined.

Individual case reports show that reinfections with SARS-CoV-2 are occurring (76, 77). However, a 2,800 person study found no symptomatic re-infections over a ~118 day window (78), and a 1,246 person study observed no symptomatic re-infections over 6 months (79). We observed heterogeneity in the magnitude of adaptive immune responses to SARS-CoV-2 persisting into the immune memory phase. It is therefore possible that a fraction of the SARS-CoV-2-infected population with low immune memory would become susceptible to re-infection relatively soon. While gender and disease severity both contribute some to the heterogeneity of immune memory reported here, the source of much of the heterogeneity in immune memory to SARS-CoV-2 is unknown and worth further examination. Perhaps heterogeneity derives from low cumulative viral load or a small initial inoculum in some individuals. Nevertheless, our data show immune memory in at least three immunological compartments was measurable in ~95% of subjects 5 to 8 months PSO, indicating that durable immunity against secondary COVID-19 disease is a possibility in most individuals.

## Supplementary Material

1

## Figures and Tables

**Table 1.** Participant characteristics

	COVID-19 (n = 188)
**Age (years)**	19–81 [Median = 40, IQR = 18.75]
**Gender**	
** Male (%)**	43% (80/188)
** Female (%)**	57% (108/188)
**Race**	
** African American or Black (%)**	3% (5/188)
** Alaskan Native or American Indian (%)**	1% (1/188)
** Asian (%)**	7% (14/188)
** Native Hawaiian or Pacific Islander (%)**	0% (0/188)
** Multiracial (%)**	1% (2/188)
** Other (%)**	1% (1/188)
** Unknown (%)**	10% (19/188)
** White (%)**	78% (146/188)
**Ethnicity**	
** Hispanic or Latino (%)**	15% (28/188)
** Non-Hispanic (%)**	80% (150/188)
** Unknown (%)**	5% (10/188)
**Hospitalization status**	
** Never hospitalized (%)**	93% (174/188)
** Hospitalized (%)**	7% (13/188)
** Unknown if hospitalized (%)**	1% (1/188)
**Sample Collection Dates**	March-October 2020
**SARS-CoV-2 PCR Positivity**	
** Positive**	77% (145/188)
** Negative**	1% (2/188)
** Not performed**	20% (37/188)
** Unknown**	2% (4/188)
**Peak Disease Severity (%) [Female (F), Male (M)]**	
** Asymptomatic (score 1)**	2% (4/188) [2F, 2M]
** Mild (Non-hospitalized; Score 2–3)**	90% (170/188) [100F, 70M]
** Moderate (Hospitalized; Score 4–5)**	3% (6/188) [3F, 3M]
** Severe (Hospitalized; Score 6+)**	4% (7/188) [3F, 4M]
** Unknown**	1% (1/188) [0F, 1M]
**Days Post Symptom Onset at Collection; n = 254**	6–240 (Median 88, IQR 97.75)
**Blood Collection Frequency**	
** Multiple Time Point Donors (2–4 times)**	27% (51/188)
** Single Time Point Donors**	73% (137/188)
