# Supplementary material for: Immunological memory to SARS-CoV-2 assessed for up to eight months after infection
Source: bioRxiv. 2020 Dec 18:2020.11.15.383323. Preprint. [Version 2] doi: 10.1101/2020.11.15.383323 (PMC7805444; doi:10.1101/2020.11.15.383323)
Supplement: 1 [file NIHPP2020.11.15.383323-supplement-1.pdf]

bioRxiv preprint doi: https://doi.org/10.1101/2020.11.15.383323; this version posted December 18, 2020. The copyright holder for this preprint (which was not certified by peer review) is the author/funder, who has granted bioRxiv a license to display the preprint in perpetuity. It is made available under aCC-BY 4.0 International license.

Supplementary Materials for

Immunological memory to SARS-CoV-2 assessed for up to eight months after infection

Jennifer M. Dan*, Jose Mateus*, Yu Kato*, Kathryn M. Hastie, Esther Dawen Yu, Caterina E. Faliti, Alba Grifoni, Sydney I. Ramirez, Sonya Haupt, April Frazier, Catherine Nakao, Vamseedhar Rayaprolu, Stephen A. Rawlings, Bjoern Peters, Florian Krammer, Viviana Simon, Erica Ollmann Saphire, Davey M. Smith, Daniela Weiskopf^, Alessandro Sette^, Shane Crotty^

Correspondence to: shane@lji.org (S.C.), alex@lji.org (A.S.), daniela@lji.org (D.W.)

This PDF file includes:

Materials and Methods
Supplementary Text
Figs. S1 to S10
Tables S1 to S2

bioRxiv preprint doi: https://doi.org/10.1101/2020.11.15.383323; this version posted December 18, 2020. The copyright holder for this preprint (which was not certified by peer review) is the author/funder, who has granted bioRxiv a license to display the preprint in perpetuity. It is made available under aCC-BY 4.0 International license.

Materials and Methods

Human Subjects

The Institutional Review Boards of the University of California, San Diego (UCSD; 200236X) and the La Jolla Institute for Immunology (LJI; VD-214) approved the protocols used for blood collection for subjects with COVID-19 who donated at all sites other than Mt. Sinai. The Icahn School of Medicine at Mt. Sinai IRB approved the samples collected at this institution in New York City (IRB-16-00791). All human subjects were assessed for medical decision-making capacity using a standardized, approved assessment, and voluntarily gave informed consent prior to being enrolled in the study. Study inclusion criteria included a diagnosis of COVID-19 or suspected COVID-19, age of 18 years or greater, willingness and ability to provide informed consent. Although not a strict inclusion criterion, evidence of positive PCR-based testing for SARS-CoV-2 was requested from subjects prior to participation. 145 cases were confirmed SARS-CoV-2 positive by PCR-based testing (**Table 1**). Two subjects tested negative by SARS-CoV-2 PCR (**Table 1**). The remainder were not tested or did not have test results available for review (**Table 1**). Subjects who had a medical history and/or symptoms consistent with COVID-19, but lacked positive PCR-based testing for SARS-CoV-2 and subsequently had negative laboratory-based serologic testing for SARS-CoV-2 were then excluded; i.e., all COVID-19 cases in this study were confirmed cases by SARS-CoV-2 PCR or SARS-CoV-2 serodiagnostics, or both. Adults of all races, ethnicities, ages, and genders were eligible to participate. Study exclusion criteria included lack of willingness to participate, lack of ability to provide informed consent, or a medical contraindication to blood donation (e.g. severe anemia). Subject samples at LJI were obtained from individuals in California and at least seven other states.

Blood collection and processing methods at LJI were performed as previously described (*5*). Briefly, whole blood was collected via phlebotomy in acid citrate dextrose (ACD) serum separator tubes (SST), or ethylenediaminetetraacetic acid (EDTA) tubes and processed for peripheral blood mononuclear cells (PBMC), serum, and plasma isolation. Most donors were screened for symptoms prior to scheduling blood draws, and had to be symptom-free and approximately 3-4 weeks out from symptom onset at the time of the initial blood draw at UCSD or LJI, respectively. Samples were coded, and then de-identified prior to analysis. Other efforts to maintain the confidentiality of participants included the labeling samples with coded identification numbers. An overview of the characteristics of subjects with COVID-19 is provided in **Table 1**.

COVID-19 disease severity was scored from 0 to 10 using a numerical scoring system based on the NIH ordinal scale (*5*, *80*). A categorical descriptor was applied based on this scoring system: “asymptomatic” for a score of 1, “mild” for a score of 2-3, “moderate” for a score of 4-5, and “severe” for a score of 6 or more. Subjects with a numerical score of 4 or higher required hospitalization (including admission for observation) for management of COVID-19. Only one of 13 hospitalized subjects is shared from the previous study of acute COVID-19 (*5*). The days PSO was determined based on the difference between the date of the blood collection and the date of first reported symptoms consistent with COVID-19. For asymptomatic subjects, the day from first positive SARS-CoV-2 PCR-based testing was used in place of the date of first reported COVID-19 symptoms.

Recombinant Proteins

Stabilized Spike protein (2P, (*81*)) and the receptor binding domain (RBD) were expressed in HEK293F cells. Briefly, DNA expressing stabilized spike protein and RBD were subcloned into separate phCMV vectors and transfected into HEK293F cells at a ratio of 1mg of DNA to 1L of

bioRxiv preprint doi: https://doi.org/10.1101/2020.11.15.383323; this version posted December 18, 2020. The copyright holder for this preprint (which was not certified by peer review) is the author/funder, who has granted bioRxiv a license to display the preprint in perpetuity. It is made available under aCC-BY 4.0 International license.

cells. The cells were cultured at 37C in a shaker incubator set to 125rpm, 80% humidity and 8% CO_2. When cell viability dropped below 80% (typically 4-5 days), media was harvested and centrifuged to remove cells. Biolock reagent was added to the supernatant media to remove any excess biotin. The media was then filtered through a 0.22um filter to remove Biolocked-aggregates. Proteins were purified using Streptrap HP 5mL columns (Cytiva) using 100mM Tris, 100mM NaCl as the Wash Buffer and 100mM Tris, 100mM NaCl, 2.5mM d-Desthiobiotin as the Elution Buffer. The eluted fractions for Spike proteins were concentrated on 100kDa Amicon filters while the RBD were concentrated on 10kDa filters. The samples were further purified using S6increase columns for the spike variants and S200increase column for RBD.

SARS-CoV-2 ELISAs

SARS-CoV-2 ELISAs were performed as previously described (*2*, *5*, *82*). Briefly, Corning 96-well half area plates (ThermoFisher 3690) were coated with 1µg/mL of antigen overnight at 4°C. Antigens included recombinant SARS-CoV-2 RBD protein, recombinant Spike protein, and recombinant Nucleocapsid protein (GenScript Z03488) (Recombinant nucleocapsid antigens were also tested from Sino Biological (40588-V07E) and Invivogen (his-sars2-n) and yielded comparable results to GenScript nucleocapsid). The following day, plates were blocked with 3% milk in phosphate buffered saline (PBS) containing 0.05% Tween-20 for 1.5 hours at room temperature. Plasma was heat inactivated at 56°C for 30-60 minutes. Plasma was diluted in 1% milk containing 0.05% Tween-20 in PBS starting at a 1:3 dilution followed by serial dilutions by 3 and incubated for 1.5 hours at room temperature. Plates were washed 5 times with 0.05% PBS-Tween-20. Secondary antibodies were diluted in 1% milk containing 0.05% Tween-20 in PBS. For IgG, anti-human IgG peroxidase antibody produced in goat (Sigma A6029) was used at a 1:5,000 dilution. For IgA, anti-human IgA horseradish peroxidase antibody (Hybridoma Reagent Laboratory HP6123-HRP) was used at a 1:1,000 dilution. The HP6123 monoclonal anti-IgA was used because of its CDC and WHO validated specificity for human IgA1 and IgA2 and lack of crossreactivity with non-IgA isotypes (*82*).

Endpoint titers were plotted for each sample, using background subtracted data. Negative and positive controls were used to standardize each assay and normalize across experiments. A positive control standard was created by pooling plasma from 6 convalescent COVID-19 donors to normalize between experiments. The limit of detection (LOD) was defined as 1:3 for IgG, 1:10 for IgA. Limit of sensitivity (LOS) for SARS-CoV-2 infected individuals was established based on uninfected subjects, using plasma from normal healthy donors never exposed to SARS-CoV-2. For cross-sectional analyses, modeling for the best fit curve (e.g., one phase decay versus simple linear regression) was performed using GraphPad Prism 8.0. Best curve fit was defined by an extra sum-of-squares F Test, selecting the simpler model unless $P < 0.05$ (*83*). Continuous decay (linear regression), one-phased decay, or two-phased decay of log data were assessed in all cases, with the best fitting statistical model chosen based on the F test; in several cases a quadratic equation fit was also considered. To calculate the $t_{1/2}$, $\log_2$ transformed data was utilized. Using the best fit curve, either a one phase decay non-linear fit or a simple linear regression (continuous decay) was utilized. For simple linear regressions, Pearson R was calculated for correlation using $\log_2$ transformed data. For one phase decay non-linear fit, R was reported. For longitudinal samples, a simple linear regression was performed, with $t_{1/2}$ calculated from $\log_2$ transformed data for each pair. For gender analyses, modeling and $t_{1/2}$ was performed similar to cross-sectional analyses; ANCOVA (VassarStats or GraphPad Prism 8.4) was then performed between male and female

bioRxiv preprint doi: https://doi.org/10.1101/2020.11.15.383323; this version posted December 18, 2020. The copyright holder for this preprint (which was not certified by peer review) is the author/funder, who has granted bioRxiv a license to display the preprint in perpetuity. It is made available under aCC-BY 4.0 International license.

data sets. ANCOVA p-values of the adjusted means were reported and considered significant if the test for homogeneity of regressions was not significant.

Neutralizing antibody assays

The pseudovirus neutralizing antibody assay was performed as previously described (*5*). Briefly, Vero cells were seeded in 96-well plates to produce a monolayer at the time of infection. Pre-titrated amounts of rVSV-SARS-Cov-2 (phCMV3-SARS-CoV-2 Spike SARS-CoV-2-pseduotyped VSV-ΔG-GFP were generated by transfecting HEK293T cells, ATCC CRL-3216) were incubated with serially diluted human plasma at 37°C for 1 hour before addition to confluent Vero cell monolayers (ATCC CCL-81) in 96-well plates. Cells were incubated for 12-16 hours at 37°C in 5% CO_2. Cells were then fixed in 4% paraformaldehyde, stained with 1µg/mL Hoechst, and imaged using a CellInsight CX5 imager to quantify the total number of cells expressing GFP. Infection was normalized to the average number of cells infected with rVSV-SARS-CoV-2 incubated with normal human plasma. The limit of detection (LOD) was established as < 1:20 based on plasma samples from a series of unexposed control subjects. Negative signals were set to 1:19. Neutralization IC50 titers were calculated using One-Site Fit LogIC50 regression in GraphPad Prism 8.0.

Detection of antigen-specific memory B cells

To detect SARS-CoV-2 specific B cells, biotinylated protein antigens were individually multimerized with fluorescently labeled streptavidin at 4°C for one hour. Full-length SARS-CoV-2 Spike (2P-stabilized, double Strep-tagged) and RBD were generated in-house. Biotinylation was performed using biotin-protein ligase standard reaction kit (Avidity, Cat# Bir500A) following the manufacturer's standard protocol and dialyzed overnight against PBS. Biotinylated Spike was mixed with streptavidin BV421 (BioLegend, Cat# 405225) and streptavidin Alexa Fluor 647 (Thermo Fisher Scientific, Cat# S21374) at 20:1 ratio (~6:1 molar ratio). Biotinylated RBD was mixed with streptavidin PE/Cyanine7 (BioLegend, Cat# 405206) at 2.2:1 ratio (~4:1 molar ratio). Biotinylated SARS-CoV-2 full length Nucleocapsid (Avi- and His-tagged; Sino Biological, Cat# 40588-V27B-B) was multimerized using streptavidin PE (BioLegend, Cat# 405204) and streptavidin BV711 (BioLegend, Cat# 405241) at 5.5:1 ratio (~6:1 molar ratio). Streptavidin PE/Cyanine5.5 (Thermo Fisher Scientific, Cat# SA1018) was used as a decoy probe to gate out SARS-CoV-2 non-specific streptavidin-binding B cells. The antigen probes prepared individually as above were then mixed in Brilliant Buffer (BD Bioscience, Cat# 566349) containing 5µM free d-biotin (Avidity, Cat# Bir500A). Free d-biotin ensured minimal cross-reactivity of antigen probes. ~10^7 previously frozen PBMC samples were prepared in U-bottom 96-well plates and stained with 50µL antigen probe cocktail containing 100ng Spike per probe (total 200ng), 27.5ng RBD, 40ng Nucleocapsid per probe (total 80ng) and 20ng streptavidin PE/Cyanine5.5 at 4°C for one hour to ensure maximal staining quality before surface staining with antibodies as listed in Table S1 was performed in Brilliant Buffer at 4°C for 30min. Dead cells were stained using LIVE/DEAD Fixable Blue Stain Kit (Thermo Fisher Scientific, Cat# L34962) in DPBS at 4°C for 30min. ~80% of antigen-specific memory (IgD^- and/or $CD27^+$) B cells detected using this method were IgM^+, IgG^+, or IgM^- IgG^- IgA^+, which were comparable to non-specific memory B cells. Based on these observations, we concluded that the antigen probes did not significantly impact the quality of surface immunoglobulin staining. Stained PBMC samples were acquired on Cytek Aurora and analyzed using FlowJo10.7.1 (BD Bioscience).

bioRxiv preprint doi: https://doi.org/10.1101/2020.11.15.383323; this version posted December 18, 2020. The copyright holder for this preprint (which was not certified by peer review) is the author/funder, who has granted bioRxiv a license to display the preprint in perpetuity. It is made available under aCC-BY 4.0 International license.

The frequency of antigen-specific memory B cells was expressed as a percentage of total B cells ($CD19^+$ $CD20^+$ $CD38^{int/-}$, $CD3^-$, $CD14^-$, $CD16^-$, $CD56^-$, LIVE/DEAD$^-$, lymphocytes), or as number per 10^6 PBMC (LIVE/DEAD$^-$ cells). LOD was set based on median + 2× standard deviation (SD) of [1 / (number of total B cells recorded)] or median + 2×SD of [10^6 / (number of PBMC recorded)]. LOS was set as the median + 2×SD of the results in unexposed donors. Phenotype analysis of antigen-specific B cells was performed only in subjects with at least 10 cells detected in the respective antigen-specific memory B cell gate. In each experiment, PBMC from a known positive control (COVID-19 convalescent subject) and unexposed subjects were included to ensure consistent sensitivity and specificity of the assay. For each data set, second order polynomial, simple linear regression, and pseudo-first order kinetic models were considered. The model with a lower Akaike's Information Criterion value was determined to be a better-fit and visualized.

<u>Activation induced markers (AIM) T cell assay</u>

Antigen-specific $CD4^+$ T cells were measured as a percentage of AIM^+ ($OX40^+CD137^+$) $CD4^+$ T and ($CD69^+CD137^+$) $CD8^+$ T cells after stimulation of PBMC with overlapping peptide megapools (MP) spanning the entire SARS-CoV-2 ORFeome, as previously described (*2*). Cells were cultured for 24 hours in the presence of SARS-CoV-2 specific MPs [1 μg/mL] or 5 μg/mL phytohemagglutinin (PHA, Roche) in 96-wells U-bottom plates at $1x10^6$ PBMC per well. A stimulation with an equimolar amount of DMSO was performed as a negative control, PHA, and stimulation with a combined $CD4^+$ and $CD8^+$ cytomegalovirus epitope MP (CMV, 1 μg/mL) were included as positive controls. Any sample with low PHA signal was excluded as a quality control.

Antigen-specific $CD4^+$ and $CD8^+$ T cells were measured as background (DMSO) subtracted data, with a minimal DMSO level set to 0.005%. All positive ORFs (> 0.02% for $CD4^+$, > 0.05% for $CD8^+$) were then aggregated into a combined sum of SARS-CoV-2-specific $CD4^+$ or $CD8^+$ T cells. The threshold for positivity for antigen-specific $CD4^+$ T cell responses (0.03%) and antigen-specific $CD8^+$ T cell responses (0.12%) was calculated using the median two-fold standard deviation of all negative controls measured (>150). The antibody panel utilized in the ($OX40^+CD137^+$) $CD4^+$ T and ($CD69^+CD137^+$) $CD8^+$ T cells AIM staining is shown in Table S2. A consistency analysis was performed for multiple measurements of AIM T cell assays by two different operators. Before merging, we compared the protein immunodominance, total SARS-CoV-2-specific $CD4^+$ and $CD8^+$ T cell responses, and half-life calculations between the two groups of experimental data. In longitudinal analyses, half-life calculations excluded any samples that were negative at both timepoints (since a half-life could not be calculated), though all data were included in the graphs.

For surface $CD40L^+OX40^+$ $CD4^+$ T cell AIM assays, experiments were performed as previously described (*5*), with the following modifications. Cells were cultured in complete RPMI containing 5% human AB serum (Gemini Bioproducts), beta-mercaptoethanol, Penicillin/Streptomycin, sodium pyruvate (NaPy), and non-essential amino acids. Prior to addition of peptide MPs, cells were blocked at 37°C for 15 minutes with 0.5μg/mL anti-CD40 mAb (Miltenyi Biotec). A stimulation with an equimolar amount of DMSO was performed to determine background subtraction, and activation from Staphylococcal enterotoxin B (SEB) at 1 μg/mL was used as (positive) quality control. LOD for antigen-specific cT_{FH} among $CD4^+$ T cells was based on the LOD for antigen-specific $CD4^+$ T cells (described above) multiplied by the average % cT_{FH} in the bulk CD4 T cells among control samples. An inclusion threshold of ten events after the cT_{FH}

bioRxiv preprint doi: https://doi.org/10.1101/2020.11.15.383323; this version posted December 18, 2020. The copyright holder for this preprint (which was not certified by peer review) is the author/funder, who has granted bioRxiv a license to display the preprint in perpetuity. It is made available under aCC-BY 4.0 International license.

CXCR5$^+$ gate was used for PD-1^{hi} and CCR6$^+$ calculations, and Mann-Whitney nonparametric and Wilcoxon signed-rank statistical tests were applied for the respective comparisons.

bioRxiv preprint doi: https://doi.org/10.1101/2020.11.15.383323; this version posted December 18, 2020. The copyright holder for this preprint (which was not certified by peer review) is the author/funder, who has granted bioRxiv a license to display the preprint in perpetuity. It is made available under aCC-BY 4.0 International license.

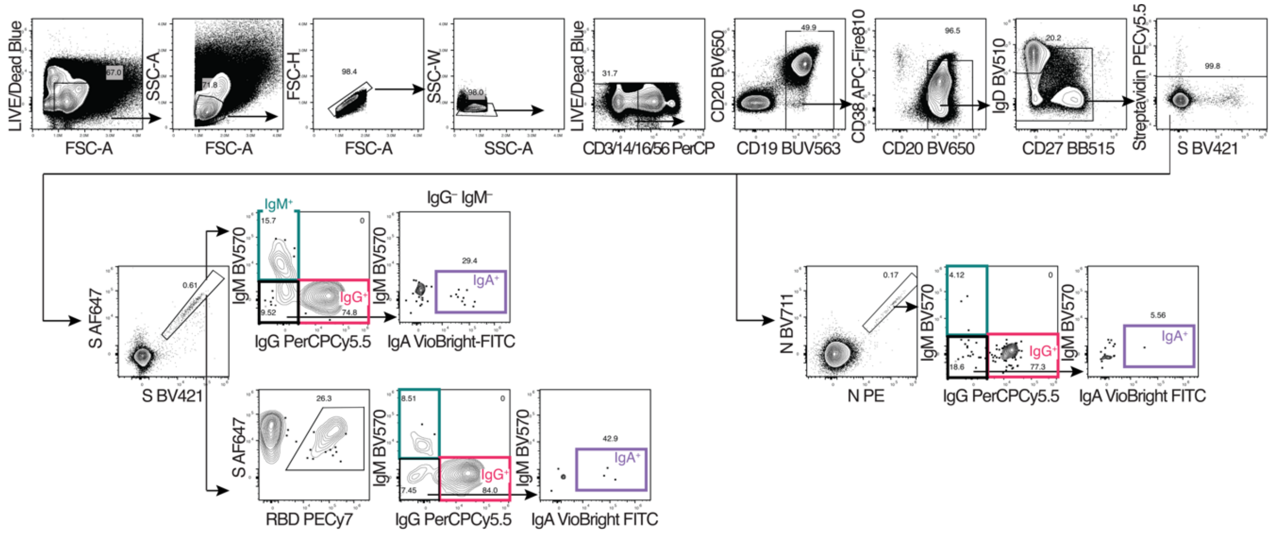

Fig. S1. SARS-CoV-2 memory B cells.

(**A**) Gating strategies to define Spike-, RBD-, or Nucleocapsid-specific memory B cells. S = SARS-CoV-2 Spike trimer. N = SARS-CoV-2 Nucleocapsid.

bioRxiv preprint doi: https://doi.org/10.1101/2020.11.15.383323; this version posted December 18, 2020. The copyright holder for this preprint (which was not certified by peer review) is the author/funder, who has granted bioRxiv a license to display the preprint in perpetuity. It is made available under aCC-BY 4.0 International license.

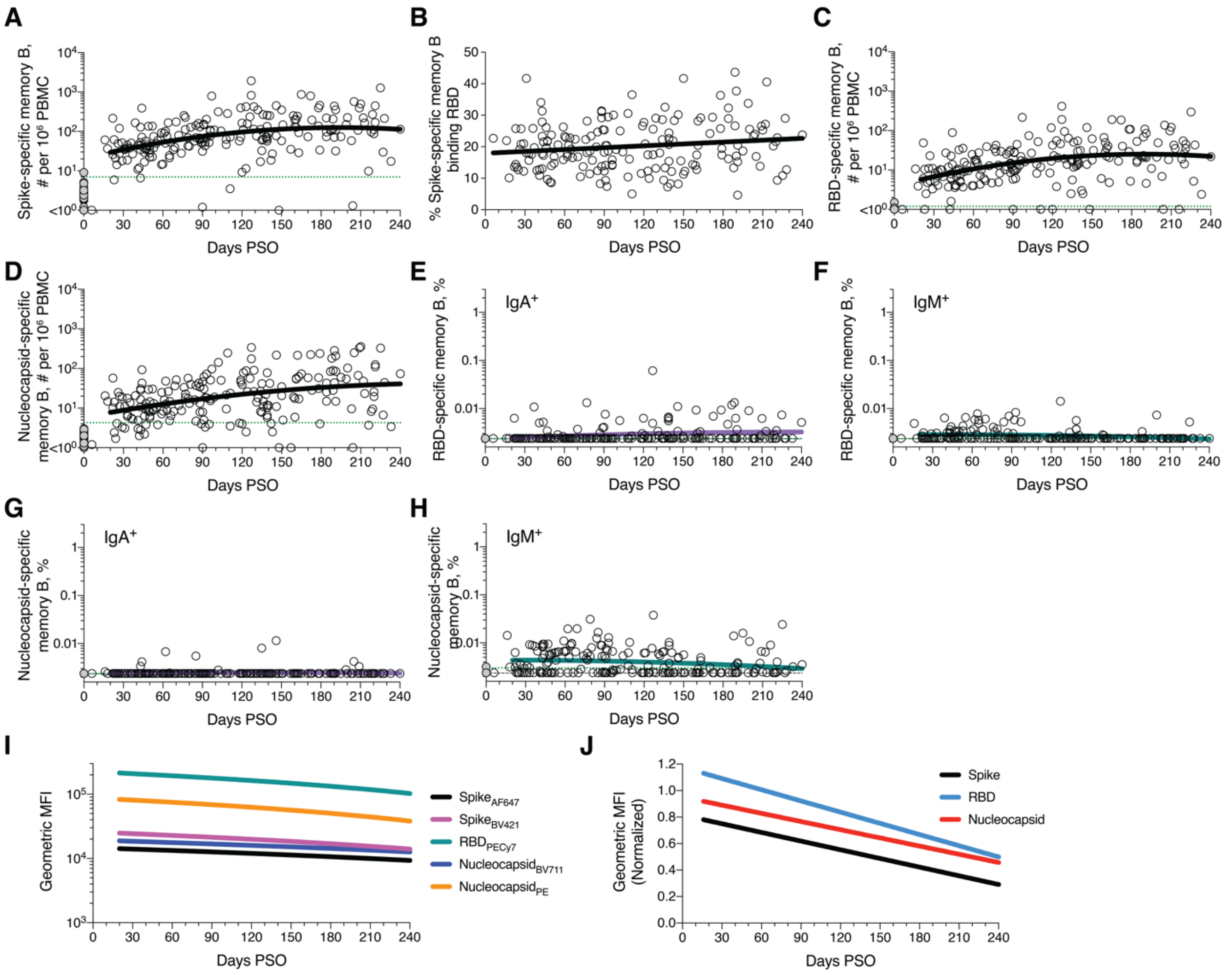

Fig. S2. Kinetics of memory B cell responses.

(**A**) Cross-sectional analysis of SARS-CoV-2 Spike-specific memory B cell numbers per 10^6 PBMC. Second order polynomial model for best fit curve (|R| = 0.38). **(B)** Percentage of Spike-specific B cells binding RBD. Simple linear regression (R = 0.15) (**C**) Cross-sectional analysis of RBD-specific memory B cell numbers per 10^6 PBMC. Second order polynomial model for best fit curve (|R| = 0.39). (**D**) Cross-sectional analysis of Nucleocapsid-specific memory B cell numbers per 10^6 PBMC. Second order polynomial model for best fit curve (|R| = 0.38). (**E**) Cross-sectional analysis of frequency (% of $CD19^+$ $CD20^+$ B cells) of RBD-specific IgA^+ memory B cells. Second order polynomial model for best fit curve (|R| = 0.19). (**F**) Cross-sectional analysis of frequency of RBD-specific IgM^+ memory B cells. Second order polynomial model for best fit curve (|R| = 0.18). (**G**) Cross-sectional analysis of frequency of SARS-CoV-2 Nucleocapsid-specific IgA^+ memory B cells. Second order polynomial model (|R| = 0.06). (**H**) Cross-sectional analysis of frequency of Nucleocapsid-specific IgM^+ memory B cells. Second order polynomial (|R| = 0.17). (**I**) Cross-sectional analysis of geometric mean fluorescence intensity of Spike, RBD and Nucleocapsid probes on S-, RBD- and Nucleocapsid-specific memory B cells, respectively. Data shown are simple linear-regression lines. (**J**) Cross-sectional analysis of geometric mean fluorescence intensity of Spike, RBD and Nucleocapsid probes on S-, RBD- and Nucleocapsid-specific memory B cells, respectively, normalized to a positive control sample. Data shown are simple linear-regression lines.

bioRxiv preprint doi: https://doi.org/10.1101/2020.11.15.383323; this version posted December 18, 2020. The copyright holder for this preprint (which was not certified by peer review) is the author/funder, who has granted bioRxiv a license to display the preprint in perpetuity. It is made available under aCC-BY 4.0 International license.

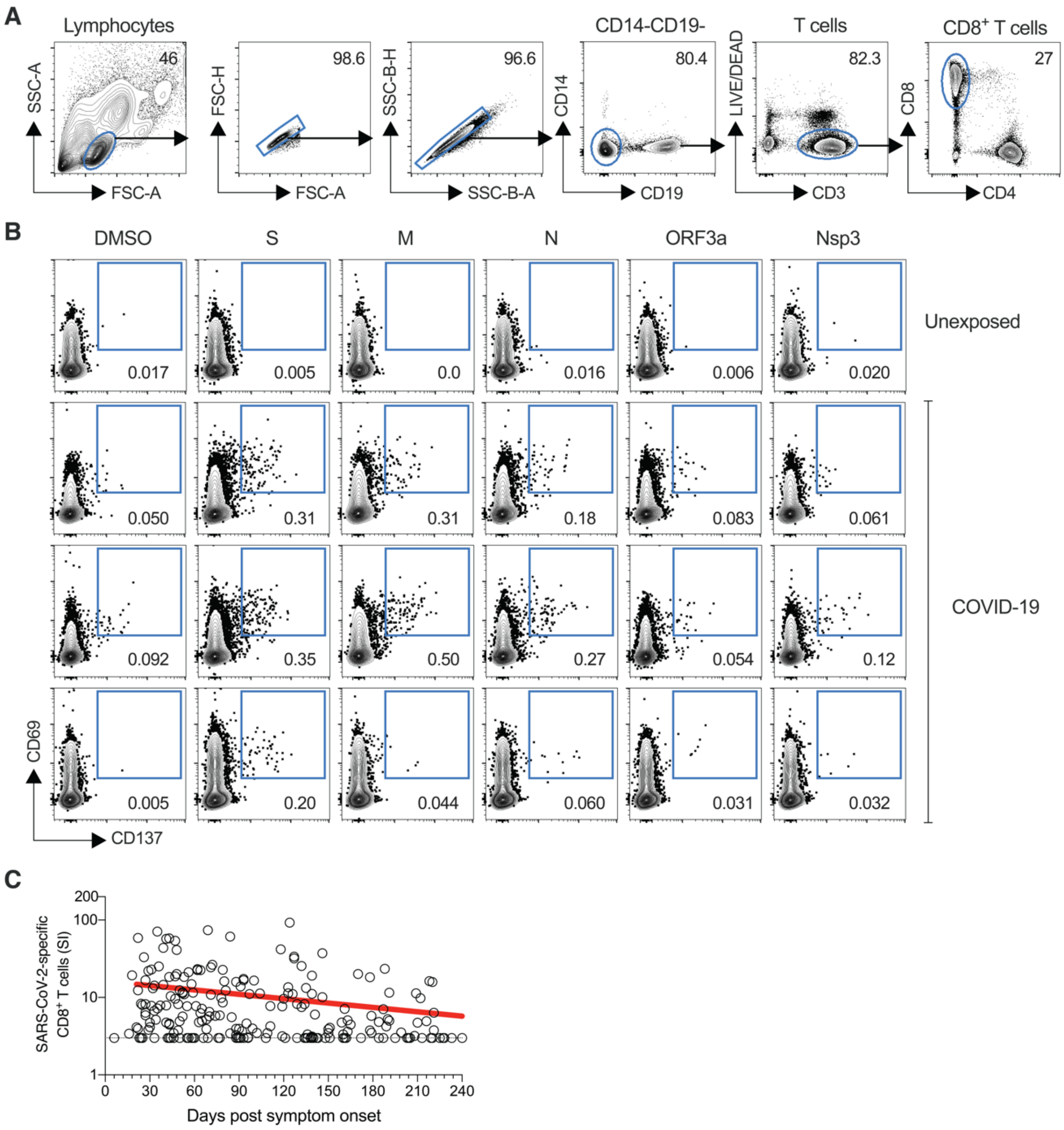

Fig. S3. SARS-CoV-2 circulating memory CD8⁺ T cells.

(A) Gating strategies to define SARS-CoV-2-specific CD8^{+} T cells by AIM assay, using individual SARS-CoV-2 ORF peptide pools. **(B)** Representative examples of flow cytometry plots of SARS-CoV-2-specific CD8^{+} T cells (CD69^{+} CD137^{+}) after overnight stimulation with Spike (S), Membrane (M), Nucleocapsid (N), ORF3a, or nsp3 peptide pools, compared to negative control stimulation (DMSO) from three COVID-19 subjects and one uninfected control. **(C)** Cross-sectional analysis of total SARS-CoV-2-specific CD8^{+} T cells, as per Figure 3, but graphing stimulation index (SI).

bioRxiv preprint doi: https://doi.org/10.1101/2020.11.15.383323; this version posted December 18, 2020. The copyright holder for this preprint (which was not certified by peer review) is the author/funder, who has granted bioRxiv a license to display the preprint in perpetuity. It is made available under aCC-BY 4.0 International license.

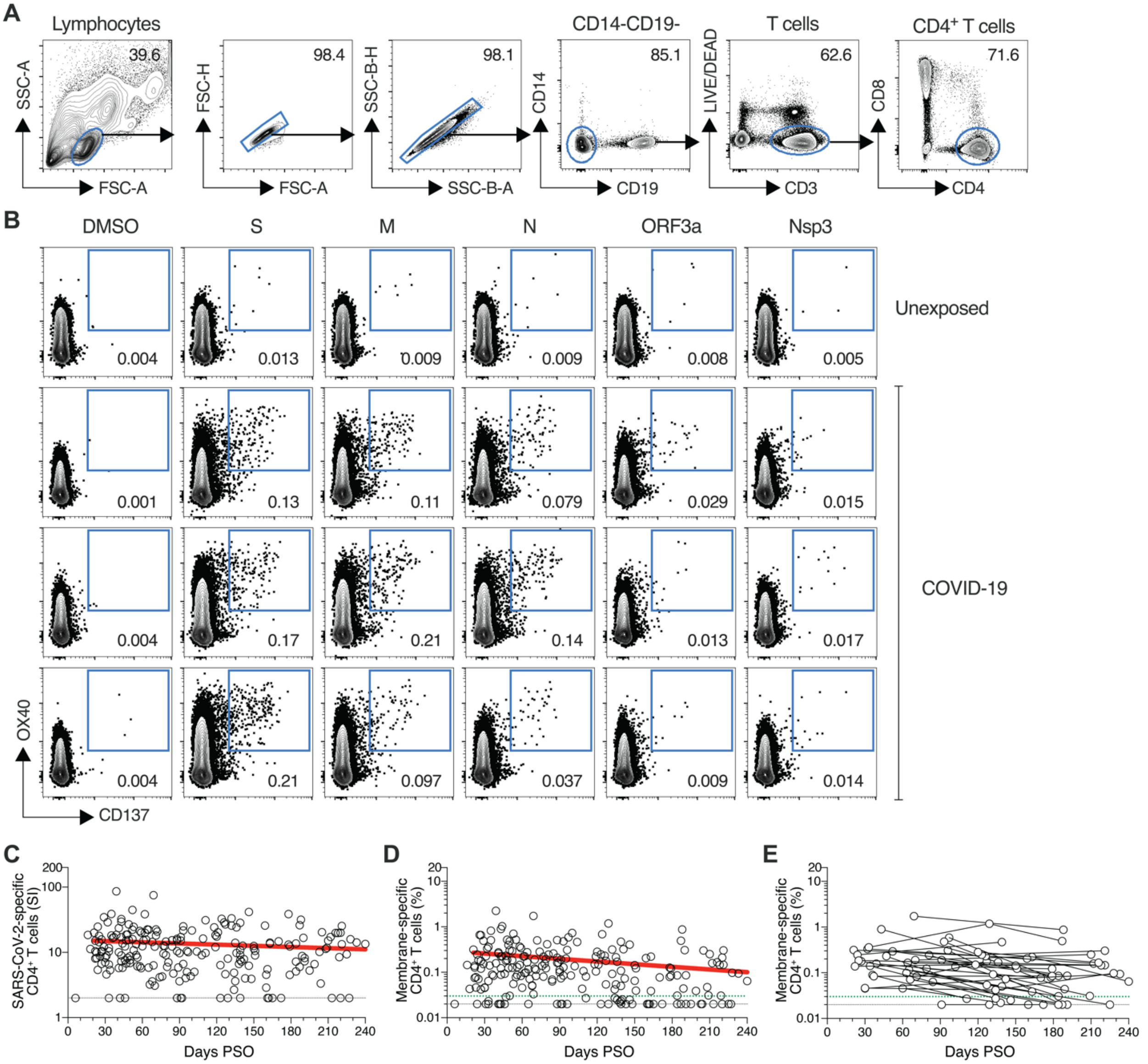

Fig. S4. SARS-CoV-2 circulating memory CD4$^+$ T cells.

(A) Gating strategies to define SARS-CoV-2-specific CD4$^+$ T cells by AIM assay, using individual SARS-CoV-2 ORF peptide pools. **(B)** Representative examples of flow cytometry plots of SARS-CoV-2-specific CD4$^+$ T cells (OX40$^+$ CD137$^+$, after overnight stimulation with Spike, M, Nucleocapsid, ORF3a, or nsp3 peptide pools, compared to negative control (DMSO). From three COVID-19 subjects and one uninfected control. **(C)** Cross-sectional analysis of total SARS-CoV-2-specific CD4$^+$ T cells, as per Figure 4, but graphing stimulation index (SI). **(D)** Cross-sectional analysis of M-specific CD4$^+$ T cells. Linear decay preferred model, $t_{1/2}$ = 153 days. R = -0.25, p = 0.0003. **(E)** Longitudinal analysis of M-specific CD4$^+$ T cells in paired samples from the same subjects. n = 215 COVID-19 subject samples for cross-sectional analysis. n = 37 COVID-19 subjects for longitudinal analysis.

bioRxiv preprint doi: https://doi.org/10.1101/2020.11.15.383323; this version posted December 18, 2020. The copyright holder for this preprint (which was not certified by peer review) is the author/funder, who has granted bioRxiv a license to display the preprint in perpetuity. It is made available under aCC-BY 4.0 International license.

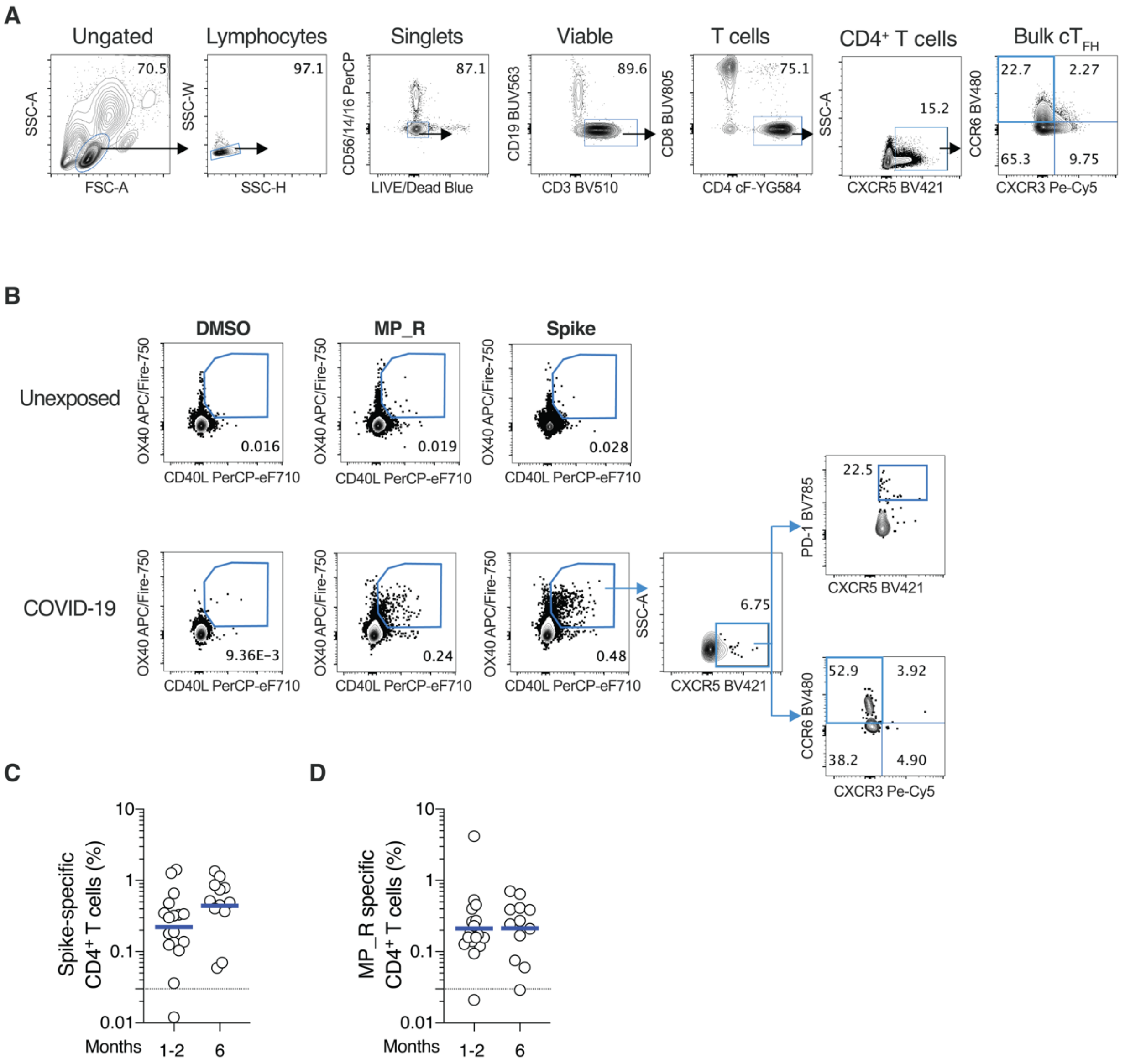

Fig. S5. SARS-CoV-2 memory T$_{FH}$ cells.

(A) Gating strategies to define SARS-CoV-2-specific CD4$^+$ T cells by AIM assay, using Spike and MP_R peptide pools. **(B)** Representative examples of flow cytometry plots of SARS-CoV-2-specific CD4$^+$ T cells. Surface CD40L$^+$OX40$^+$, after overnight stimulation with Spike or MP_R peptide pools, compared to negative control (DMSO) from a representative COVID-19 subject and an uninfected control. **(C, D)** SARS-CoV-2-specific CD4$^+$ T cells based on surface CD40L$^+$OX40$^+$, gated as in A, after overnight stimulation with Spike or MP_R peptide pools. n = 29 COVID-19 subject samples (white circles), n = 17 at 1-2 mo, n = 12 at 6 mo.

bioRxiv preprint doi: https://doi.org/10.1101/2020.11.15.383323; this version posted December 18, 2020. The copyright holder for this preprint (which was not certified by peer review) is the author/funder, who has granted bioRxiv a license to display the preprint in perpetuity. It is made available under aCC-BY 4.0 International license.

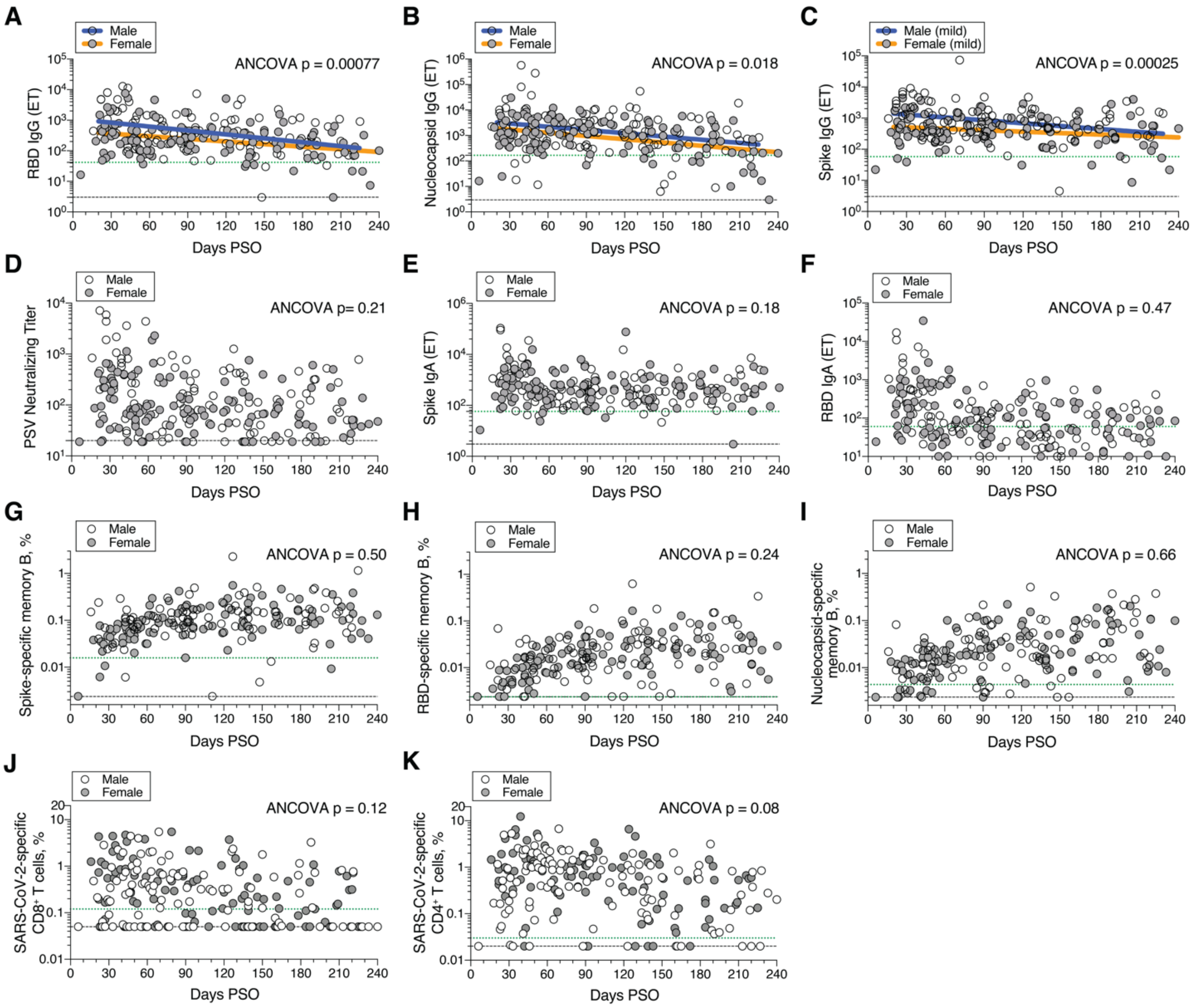

Fig. S6. Immune memory and gender.

Cross-sectional analyses of SARS-CoV-2 immune memory by male and female gender. **(A)** RBD IgG titers. Males: Continuous decay preferred model, initial $t_{1/2}$ = 24 days, R = -0.39, p<0.0001. Females: linear decay preferred model, $t_{1/2}$ = 94 days, 95% CI: 64-178 days R = -0.36, p<0.0001. ANCOVA = 0.00077, Test for homogeneity of regressions F = 1.32, p = 0.25. **(B)** Nucleocapsid IgG titers. Males: Continuous decay preferred model, $t_{1/2}$ = 70 days, 95% CI: 42-209 days, R = -0.28, p = 0.0035. Females: continuous decay preferred model, $t_{1/2}$ = 64 days, 95% CI: 47-104 days, R = -0.44, p < 0.0001. ANCOVA p = 0.018, Test for homogeneity of regressions F = 0, p = 1.0. **(C)** Spike IgG titers of non-hospitalized patients. Males: Continuous decay preferred model $t_{1/2}$ = 574 days, 95% CI: 345-1698 days, R = -0.30, p = 0.0035. Females: continuous decay preferred model, $t_{1/2}$ = 1075 days, 95% CI: 537-1,340,303 days, R = -0.19, p = 0.0502. ANCOVA p = 0.00025, Test for homogeneity of regressions F = 2.59, p = 0.11. **(D)** PSV neutralizing titers. **(E)** Spike IgA titers. **(F)** RBD IgA titers. **(G)** Frequency (% of $CD19^+$ $CD20^+$ B cells) of SARS-CoV-2 Spike-specific total (IgG^+, IgA^+, or IgM^+) memory B cells, as per Figure 2C. **(H)** Frequency of SARS-CoV-2 RBD-specific total memory B cells, as per Figure 2E. **(I)** Frequency of SARS-CoV-2 Nucleocapsid-specific total memory B cells, as per Figure 2G. **(J)**. Frequency (% of $CD8^+$ T cells) of total SARS-CoV-2-specific $CD8^+$ T cells, as per Figure 3B. **(K)** Frequency (% of $CD4^+$ T cells) of total SARS-CoV-2-specific $CD4^+$ T cells, as per Figure 4B.

bioRxiv preprint doi: https://doi.org/10.1101/2020.11.15.383323; this version posted December 18, 2020. The copyright holder for this preprint (which was not certified by peer review) is the author/funder, who has granted bioRxiv a license to display the preprint in perpetuity. It is made available under aCC-BY 4.0 International license.

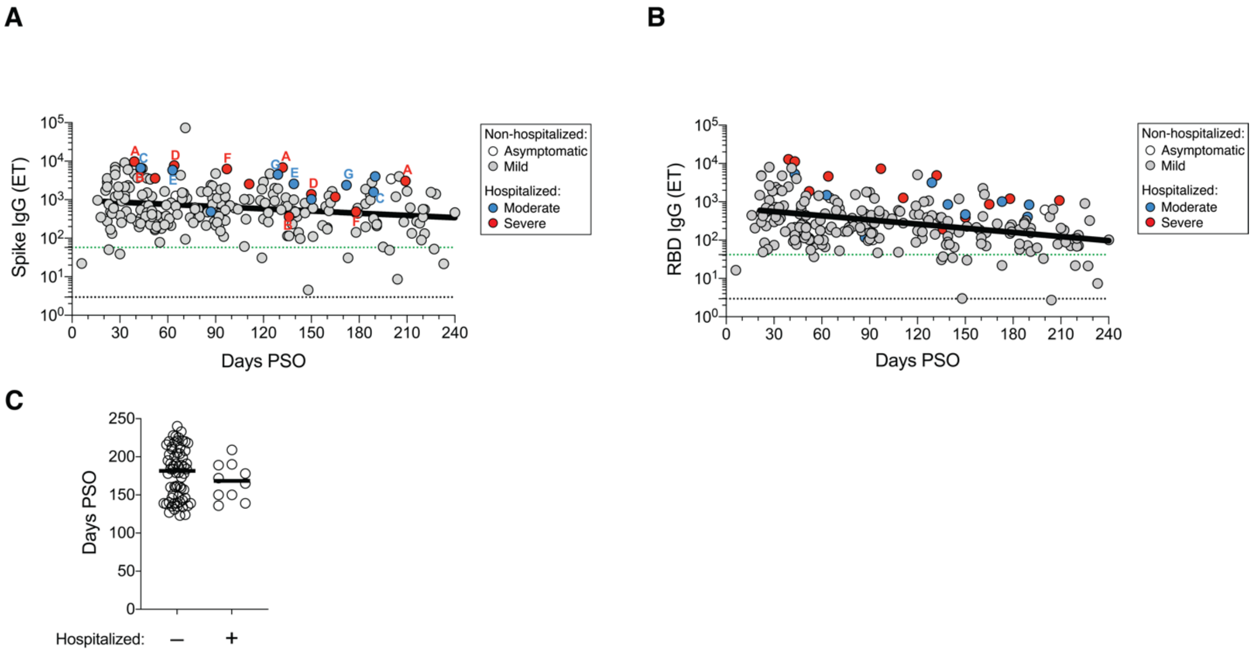

Fig. S7. Serological memory and disease severity.

(A) Cross-sectional analysis of Spike IgG, as per Figure 1A, color coded based on subject COVID-19 disease severity (white: asymptomatic, gray: mild, blue: moderate, red: severe). Letters indicate donors that were sampled at multiple timepoints after the onset of symptoms. One letter per donor. **(B)** Cross-sectional analysis of RBD IgG, as per Figure 1C, color coded based on subject COVID-19 disease severity. (C) Distribution of timepoints of COVID-19 convalescent subjects (120+ days PSO) analyzed in Figure 5B. Line indicates median. For subjects with multiple sample timepoints, only the final timepoint was used for these analyses. $p = 0.40$, Mann-Whitney test.

bioRxiv preprint doi: https://doi.org/10.1101/2020.11.15.383323; this version posted December 18, 2020. The copyright holder for this preprint (which was not certified by peer review) is the author/funder, who has granted bioRxiv a license to display the preprint in perpetuity. It is made available under aCC-BY 4.0 International license.

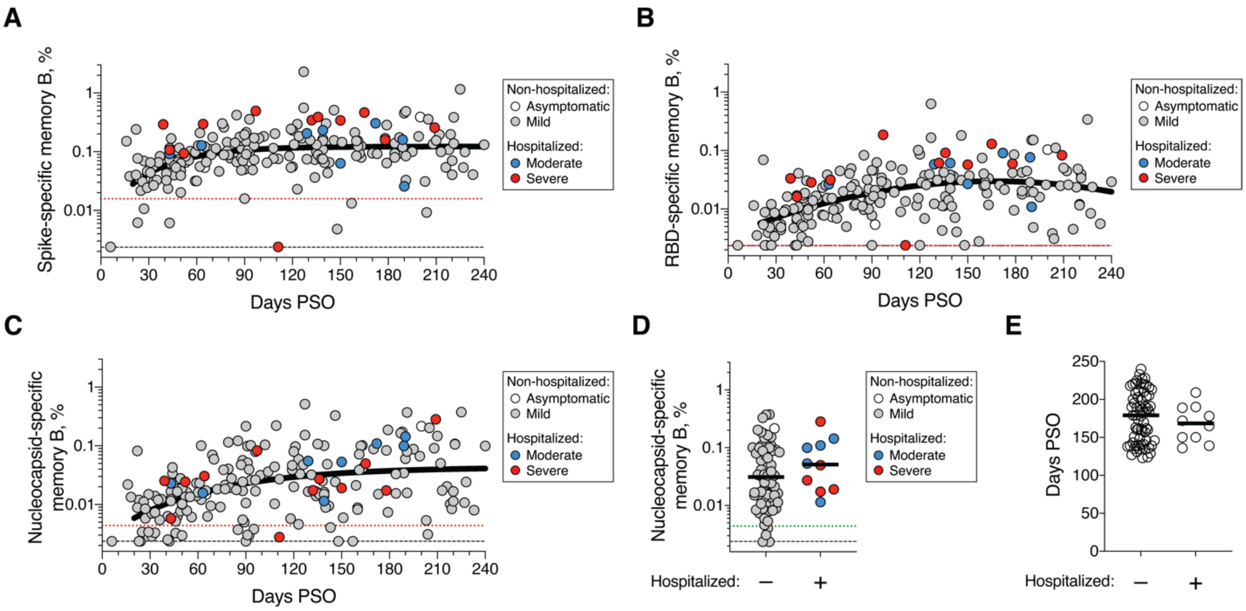

Fig. S8. Memory B cells and disease severity.

(A) Cross-sectional analysis of SARS-CoV-2 Spike-specific total (IgG^{+}, IgA^{+}, or IgA^{+}) memory B cells, as per Figure 2C, color coded based on subject COVID-19 disease severity (white: asymptomatic, gray: mild, blue: moderate, red: severe). **(B)** Cross-sectional analysis of RBD-specific total memory B cells, as per Figure 2E, color coded based on subject COVID-19 disease severity. **(C)** Cross-sectional analysis of Nucleocapsid-specific total memory B cells, as per Figure 2G, color coded based on subject COVID-19 disease severity. (**D**) Frequency of Nucleocapsid-specific memory B cells at 120+ days PSO in non-hospitalized (Asymptomatic and Mild) and hospitalized cases (Moderate and Severe). $p = 0.20$, Mann-Whitney test. **(E)** Distribution of timepoints of COVID-19 convalescent subjects (120+ days PSO) analyzed in Figure 5C, S8D. Line indicates median. For subjects with multiple sample timepoints, only the final timepoint was used for these analyses. $p = 0.47$, Mann-Whitney test.

bioRxiv preprint doi: https://doi.org/10.1101/2020.11.15.383323; this version posted December 18, 2020. The copyright holder for this preprint (which was not certified by peer review) is the author/funder, who has granted bioRxiv a license to display the preprint in perpetuity. It is made available under aCC-BY 4.0 International license.

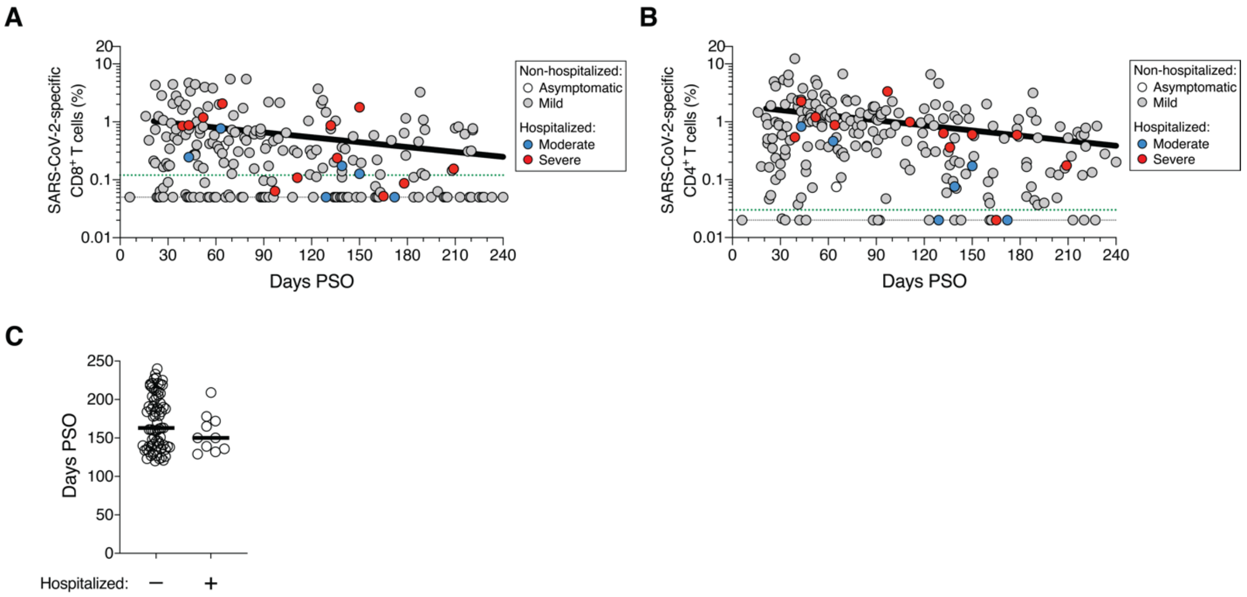

Fig. S9. T cell memory and disease severity.

(A) Cross-sectional analysis of SARS-CoV-2-specific $CD8^+$ T cells, as per Figure 3B, color coded based on subject COVID-19 disease severity (white: asymptomatic, gray: mild, blue: moderate, red: severe). **(B)** Cross-sectional analysis of SARS-CoV-2-specific $CD4^+$ T cells, as per Figure 4B, color coded based on subject COVID-19 disease severity. **(C)** Distribution of timepoints of COVID-19 convalescent subjects (120+ days PSO) analyzed in Figure 5D-E. Line indicates median. For subjects with multiple sample timepoints, only the final timepoint was used for these analyses. $p = 0.23$, Mann-Whitney test.

bioRxiv preprint doi: https://doi.org/10.1101/2020.11.15.383323; this version posted December 18, 2020. The copyright holder for this preprint (which was not certified by peer review) is the author/funder, who has granted bioRxiv a license to display the preprint in perpetuity. It is made available under aCC-BY 4.0 International license.

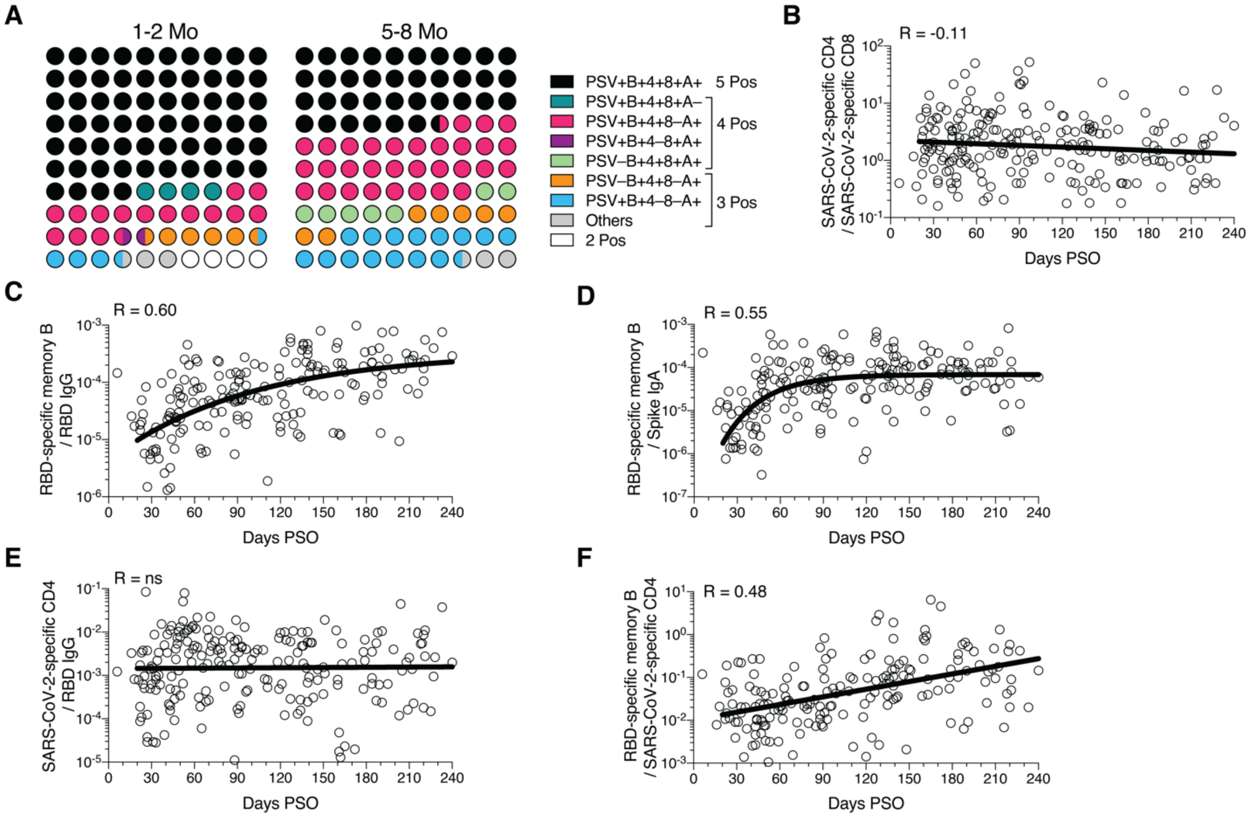

Fig. S10. Immune memory relationships.

(A) Percentage dot plots showing frequencies (normalized to 100%) of subjects with indicated immune memory components during the early (1-2 mo) or late (5-8 mo) phase. "PSV", PSV-neutralizing antibodies. "B", RBD-specific memory B cells. "4", SARS-CoV-2 specific $CD4^+$ T cells. "8", SARS-CoV-2 specific $CD8^+$ T cells. "A", Spike-specific IgA. n = 78 (1-2 mo), n = 44 (5-8 mo). **(B)** The ratio of SARS-CoV-2 specific $CD4^+$ T cell frequency relative to SARS-CoV-2 specific $CD8^+$ T cell frequency (best-fit simple linear regression line, $|R| = 0.11$). Three data points are outside the axis limits. **(C)** The ratio of RBD-specific memory B cell frequency (percentage) relative to RBD-specific IgG (pseudo-first order kinetic model, $|R| = 0.60$). Three data points are outside the axis limits. **(D)** The ratio of RBD-specific memory B cell frequency (percentage) relative to Spike IgA antibodies (pseudo-first order kinetic model, $|R| = 0.55$). One data point is outside the axis limits. **(E)** The ratio of SARS-CoV-2 specific $CD4^+$ T cell frequency relative to RBD IgG antibodies (best-fit simple linear regression line, $R = 0.046$). Three data points are outside the axis limits. **(F)** The ratio of RBD-specific memory B cell frequency (percentage) relative to total SARS-CoV-2 specific $CD4^+$ T cell frequency (best-fit simple linear regression line, $|R| = 0.48$). One data point is outside the axis limits. For Figure 5H: The ratio of RBD-specific memory B cell frequency (percentage) relative to Spike IgA antibodies (blue curve; best-fit pseudo-first order kinetic curve transformed by $\times 10^6$), RBD IgG antibodies (orange; best-fit pseudo-first order kinetic curve transformed by $\times 10^5$) and total SARS-CoV-2 specific $CD4^+$ T cell frequency purple; best-fit simple linear regression line transformed by $\times 10^2$), or the ratio of SARS-CoV-2 specific $CD4^+$ T cell frequency relative to SARS-CoV-2 specific $CD8^+$ T cell frequency (teal; best-fit simple linear regression line) and RBD IgG antibodies (black; best-fit simple linear regression line transformed by $\times 10^3$).

bioRxiv preprint doi: https://doi.org/10.1101/2020.11.15.383323; this version posted December 18, 2020. The copyright holder for this preprint (which was not certified by peer review) is the author/funder, who has granted bioRxiv a license to display the preprint in perpetuity. It is made available under aCC-BY 4.0 International license.

Table S1.
Memory B cell flow cytometry panel.

Reagents	Source	Identifier	Dilution
Mouse anti-human CD62L BV615 (clone SK11)	BD Bioscience	Cat# 565219	1:200
Mouse anti-human CD19 BUV563 (clone SJ25C1)	BD Bioscience	Cat# 612916	1:200
Mouse anti-human FCRL5 (CD307e) BUV615 (clone 509F6)	BD Bioscience	Cat# 751131	1:50
Mouse anti-human CD95 BUV737 (clone DX2)	BD Bioscience	Cat# 612790	1:200
Mouse anti-human CCR6 BUV805 (clone 11A9)	BD Biosicnece	Cat# 749361	1:200
Mouse anti-human CD138 BV480 (clone MI15)	BD Bioscience	Cat# 566140	1:50
Mouse anti-human IgD BV510 (clone IA6-2)	BioLegend	Cat# 348220	1:200
Mouse anti-human IgM BV570 (clone MHM-88)	BioLegend	Cat# 314517	1:200
Mouse anti-human CD24 BV605 (clone ML5)	BioLegend	Cat# 311124	1:200
Mouse anti-human CD20 BV650 (clone 2H7)	BioLegend	Cat# 302336	1:200
Rat anti-human CXCR5 BV750 (clone RF8B2)	BD Bioscience	Cat# 747111	1:200
Mouse anti-human CD71 BV786 (clone M-A712)	BD Bioscience	Cat# 563768	1:200
Mouse anti-human CD27 BB515 (clone M-T271)	BD Bioscience	Cat# 564642	1:200
Mouse anti-human IgA Vio Bright FITC (clone IS11-8E10)	Miltenyi Biotec	Cat# 130-113-480	1:400
Mouse anti-human CD3 PerCP (clone SK7)	BioLegend	Cat# 344814	1:100
Mouse anti-human CD14 PerCP (clone 63D3)	BioLegend	Cat# 367152	1:200
Mouse anti-human CD16 PerCP (clone 3G8)	BioLegend	Cat# 302030	1:200
Mouse anti-human CD56 PerCP (clone HCD56)	BioLegend	Cat# 318342	1:200
Rat anti-human IgG PerCP/Cyanine5.5 (clone M1310G05)	BioLegend	Cat# 410710	1:100
Mouse anti-human CD85j PE/Dazzle 594	BioLegend	Cat# 333716	1:100
Mouse anti-human CD11c PE/Cyanine5 (clone 3.9)	BioLegend	Cat# 301610	1:200
Mouse anti-human CD21 Alexa Fluor 700 (clone Bu32)	BioLegend	Cat# 354918	1:50
LIVE/DEAD Fixable Blue Stain Kit	ThermoFisher	Cat# L34962	1:400

bioRxiv preprint doi: https://doi.org/10.1101/2020.11.15.383323; this version posted December 18, 2020. The copyright holder for this preprint (which was not certified by peer review) is the author/funder, who has granted bioRxiv a license to display the preprint in perpetuity. It is made available under aCC-BY 4.0 International license.

Table S2.
Antibodies utilized in the CD8$^+$ and CD4$^+$ T cell activation induced markers (AIM) assays

Reagents	Source	Identifier	Dilution
Mouse anti-human CD45RA BV421 (clone HI100)	BioLegend	Cat# 304130	1:50
Mouse anti-human CD14 BUV563 (clone M5E2)	BD Bioscience	Cat# 741360	1:100
Mouse anti-human CD19 BUV805 (clone HIB19)	BD Bioscience	Cat# 742007	1:100
Fixable Viability Dye eFluor 506	ThermoFisher	Cat# 65-0866-18	1:200
Mouse anti-human CD8a BV650 (clone RPA-T8)	BioLegend	Cat# 301042	1:50
Mouse anti-human CD4 BV605 (clone RPA-T4)	BD Bioscience	Cat# 562658	1:25
Mouse anti-human CCR7 FITC (clone G043H7)	BioLegend	Cat# 353216	1:50
Mouse anti-human CD69 PE (clone FN50)	BD Bioscience	Cat# 555531	1:10
Mouse anti-human OX40 PECy7 (clone Ber-ACT35)	BioLegend	Cat# 350012	1:50
Mouse anti-human CD137 APC (clone 4B4-1)	BioLegend	Cat# 309810	1:25
Mouse anti-human CD3 AF700 (clone UCHT1)	ThermoFisher	Cat# 56-0038-42	1:25
Mouse anti-human CD62L BV615 (clone SK11)	BD Bioscience	Cat# 565219	1:200
Mouse anti-human CD19 BUV563 (clone SJ25C1)	BD Bioscience	Cat# 612916	1:200
